# Toll-Like Receptor 7 Mediates Inflammation Resolution and Inhibition of Angiogenesis in Non-Small Cell Lung Cancer

**DOI:** 10.3390/cancers13040740

**Published:** 2021-02-10

**Authors:** Federica Liotti, Maria Marotta, Daniela Sorriento, Emanuela Pone, Francesco Morra, Rosa Marina Melillo, Nella Prevete

**Affiliations:** 1 Institute of Experimental Endocrinology and Oncology (IEOS), CNR, 80131 Naples, Italy; federica.liotti@unina.it (F.L.); francesco_morra@hotmail.it (F.M.); 2 Department of Molecular Medicine and Medical Biotechnology, University of Naples Federico II, 80131 Naples, Italy; maria.m_1994@libero.it (M.M.); emanuela.pone@unina.it (E.P.); 3 Department of Advanced Biomedical Sciences, University of Naples Federico II, 80131 Naples, Italy; daniela.sorriento@unina.it; 4 Department of Translational Medical Sciences, University of Naples Federico II, 80131 Naples, Italy

**Keywords:** resolution of inflammation, toll-like receptor 7, angiogenesis, lung cancer

## Abstract

**Simple Summary:**

The progression of cancer is strictly linked to the formation of new blood vessels responsible for nutrition supply of the tumor. We identified TLR7 as an inhibitor of lung cancer vascularization. TLR7 is part of a large family of immune receptors that function as “sensors” of pathogen- and damage-derived signals. We found that TLR7 exerts antitumor functions in non-small cell lung cancer by inducing the production of specific molecules with inhibitory properties against new blood vessel formation. These molecules are known as specialized pro-resolving mediators (SPMs) and are derived from ω-3 and ω-6 fatty acids. We believe that the results obtained suggest novel potential targets and strategies to treat lung cancer.

**Abstract:**

Pattern recognition receptors (PRR) promote inflammation but also its resolution. We demonstrated that a specific PRR—formyl peptide receptor 1 (FPR1)—sustains an inflammation resolution response with anti-angiogenic and antitumor potential in gastric cancer. Since toll-like receptor 7 (TLR7) is crucial in the physiologic resolution of airway inflammation, we asked whether it could be responsible for pro-resolving and anti-angiogenic responses in non-small cell lung cancer (NSCLC). TLR7 correlated directly with pro-resolving and inversely with angiogenic mediators in NSCLC patients, as revealed by a publicly available RNAseq analysis. In NSCLC cells, depletion of TLR7 caused an upregulation of angiogenic mediators and a stronger vasculogenic response of endothelial cells compared to controls, assessed by qPCR, ELISA, protein array, and endothelial cell responses. TLR7 activation induced the opposite effects. TLR7 silencing reduced, while its activation increased, the pro-resolving potential of NSCLC cells, evaluated by qPCR, flow cytometry, and EIA. The increased angiogenic potential of TLR7-silenced NSCLC cells is due to the lack of pro-resolving mediators. MAPK and STAT3 signaling are responsible for these activities, as demonstrated through Western blotting and inhibitors. Our data indicate that TLR7 sustains a pro-resolving signaling in lung cancer that inhibits angiogenesis. This opens new possibilities to be exploited for cancer treatment.

## 1. Introduction

Pattern recognition receptors (PRRs) are sensors of conserved pathogen- or damage-associated molecular patterns (PAMPs/DAMPs) playing a key role in innate immune response [1]. This family includes, among the others, the toll-like receptors (TLRs), the C-type lectin receptors (CLRs), the retinoic acid-inducible gene I-like receptors (RLRs), the nucleotide oligomerization domain-like receptors (NLRs), and the formyl peptide receptors (FPRs) [1,2,3,4].

PRRs are constitutively expressed on several cell types, including immune and epithelial cells [2]. They sustain immune cell recruitment and activation [2] and regulate wound healing and homeostasis of epithelia [5,6,7]. PRRs are able to initiate inflammation, but some of them can also promote its resolution, depending on the environmental context and on the specific ligand [8,9,10].

Resolution of inflammation is a process specifically activated to ensure tissue restitution to its homeostatic function [11]. The activity of lipoxygenases (ALOX5 and 15) on omega-3/6 (ω-3/6) essential polyunsaturated fatty acids (PUFAs) [11] generates specialized pro-resolving mediators (SPMs), the main molecules responsible for inflammation resolution. Consistent with their biological functions, a deficit in pro-resolving pathways has been linked to the establishment of inflammatory conditions [12], and of diseases due to tissue damage [13,14] or abnormal vascular remodeling [15,16]. A small number of studies demonstrated that resolution mechanisms can affect cancer initiation and/or progression also [10,17,18,19].

The role of PRRs in cancer cells remains largely unknown [20]. With the aim to study the role of PRRs in the context of cancer, we recently demonstrated that a specific PRR, namely FPR1, functions as a tumor suppressor in gastric cancer (GC) by activating pro-resolving pathways and suppressing angiogenesis [17,21]. Consistently, a low-activity FPR1 polymorphism has been positively associated with the increased risk of GC in humans [22,23].

Here, we investigated whether a specific PRR might regulate SPM production and angiogenesis in non-small cell lung cancer (NSCLC) also. We focused our attention on TLR7, since it has already been demonstrated that this receptor plays a crucial role in the synthesis of SPMs and in the consequent resolution of allergic airway inflammation [24].

Here we demonstrate that (i) TLR7 expression and activation inversely correlated with the angiogenic potential in NSCLC cells and patients; (ii) TLR7 expression and activation directly correlated with pro-resolving pathway components’ expression in NSCLC cells and patients; (iii) the increased angiogenic potential of NSCLC cells silenced for TLR7 can be restored by the addition of SPMs; (iv) TLR7-mediated activation of MAPK and STAT3 signaling is responsible for its pro-resolving and anti-angiogenic properties. In support of our observations, a publicly available RNAseq analysis shows that TLR7 mRNA expression levels correlated with the disease-free, progression-free, and disease-specific survival status of NSCLC patients.

Our data indicate that PRRs sustain a pro-resolving signaling in cancer cells that inhibits angiogenesis and identify TLR7 as crucial in this phenomenon in NSCLC cells.

## 2. Results

### 2.1. Increased mRNA Expression of TLR7 Associated Directly with Pro-Resolving and Inversely with Pro-Angiogenic Markers in NSCLC Patients

It has been reported that TLR7 activation enhanced the production of SPMs both in vitro and in vivo, playing a key role in promoting the physiologic resolution of airway inflammation [24]. Thus, TLR7 is a candidate regulator of inflammation resolution and inhibition of angiogenesis in NSCLC also.

We queried the publicly available cBioPortal for Cancer Genomics database (http://www.cbioportal.org (accessed on 4 January 2021)) [25,26], obtaining co-expression data on 503 NSCLC samples: mRNA levels of TLR7 significantly and directly correlated with mRNA expression levels of the pro-resolving factors ALOX5 and ALOX5 activating protein (ALOX5AP) (Figure S1A). As previously demonstrated, pro-resolving pathways exert an anti-angiogenic effect [17]. Consistently, TLR7 mRNA levels inversely correlated with mRNA levels of two key angiogenic mediators (i.e., vascular endothelial growth factor-A (VEGF-A) and angiopoietin 1 (Ang)) (Figure S1A).

Aware of the limits of the method, which does not discriminate the expression of TLR7 between different cell types (tumor or stroma), we asked whether TLR7 expression levels correlate with NSCLC patients’ clinical attributes. In accordance with our hypothesis, the Cancer Genomics database shows that TLR7 mRNA expression levels are associated with better overall survival of NSCLC patients, assessed as progression free status, disease free status, and disease-specific survival status (Figure S1B).

We found no correlation between the expression levels of various PRRs (i.e., FPR1, TLR1, TLR2, TLR3, TLR4, TLR5, TLR6, TLR8, TLR9, TLR10, NOD1, NOD2, NALP1, NALP2, NALP3, RIG1, MDA5) and clinical features of NSCLC patients (data available on http://www.cbioportal.org (accessed on 4 January 2021)).

Altogether, these data support the hypothesis that TLR7 could be the PRR responsible for the maintenance of the pro-resolving tone in lung cancer epithelial cells.

### 2.2. TLR7 Regulates the Angiogenic Potential of NSCLC Cells

We first aimed to evaluate the role of TLR7 in NSCLC cells, paying particular attention to its impact on tumor angiogenic potential in order to verify whether its role is similar to that of FPR1 in gastric cancer (GC) [17,21].

The formation of capillary-like tube structures in the extracellular matrix by endothelial cells (ECs) is a classic method to measure angiogenesis in vitro [27]. To investigate whether differences in TLR7 expression/activation control functional angiogenic properties, we studied the ability of NSCLC cell conditioned media (CM) to induce human umbilical vein endothelial cell (HUVEC) network formation on a Matrigel substrate. In particular, we evaluated tubule formation in vitro in response to CM of NSCLC cells silenced for TLR7 (shTLR7) or stimulated by imiquimod (1 μg/mL), a selective agonist to TLR7 [28,29], compared to the relative controls. To confirm that FPR1, the key modulator of these pathways in GC, does not exert a specific role in NSCLC, we also investigated the angiogenic potential of FPR1-silenced A549.

First, we verified the expression levels of TLR7 and of FPR1 on three different NSCLC cell lines (i.e., A549, H1975, and HOP62) (Figure S2A). We stably transfected A549 cells with vectors expressing short hairpin RNAs (shRNAs) targeting TLR7 (shTLR7), FPR1 (shFPR1), or with non-targeting shRNAs (shCTR). We identified clones expressing reduced levels of each receptor (Figure S2B).

**Figure 1:**
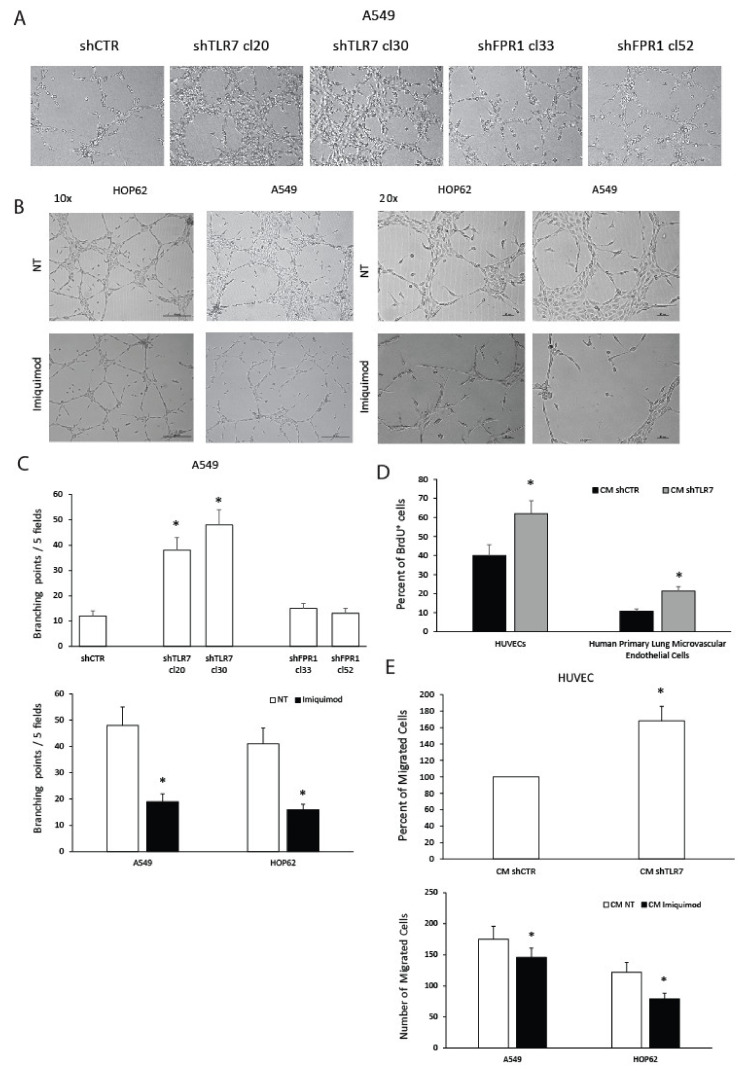
Effects of toll-like receptor 7 (TLR7) modulation in non-small cell lung cancer (NSCLC) cells on functional angiogenic responses. Human umbilical vein endothelial cells (HUVECs) were cultured in the presence of cell culture conditioned media (CM) from A549 shCTR (a mass population), shTLR7 (two clones), and shFPR1 (two clones) (**A**), or CM from A549 or HOP62 treated or untreated (NT) with imiquimod (1 μg/mL) (10× and 20× magnifications) (**B**) in a 24-well plate. After 3 days, cells were fixed with ice-cold 70% ethanol, and tubule formation was evaluated. Sample images (**A**,**B**) and a quantification of the angiogenic response (**C**) are reported. Data are represented as mean ± SD of three independent experiments. * *p* < 0.05 compared to the control. (**D**) Cell proliferation assessed as BrdU incorporation of endothelial cells (HUVEC and human primary lung microvascular endothelial cell (HPLMEC)) cultured for 36 h in CM of A549 shCTR (a mass population) and shTLR7 (2 clones). Data are represented as mean ± SD of three independent experiments. * *p* < 0.05 compared to the control. (**E**) Percent of HUVEC migrated cells toward CM from A549 shCTR (a mass population) and shTLR7 (two clones) or from A549 and HOP62 cells treated or not with imiquimod (1 μg/mL). Data are represented as mean ± SD of three independent experiments. * *p* < 0.05 compared to the control.

As shown in Figure 1A, HUVECs plated in the presence of A549 shCTR cell CM formed only a few tube structures in 3 days. On the other hand, when the cells were plated in the presence of A549 shTLR7 CM, a significantly increased number of formed tube structures compared to that for shCTR CM was observed (Figure 1A,C). shFPR1 A549 cells displayed an angiogenic potential comparable to that of A549 shCTR cells (Figure 1A,C), confirming that FPR1 is not crucial in the modulation of the angiogenic response of NSCLC cells.

We then evaluated the ability of TLR7 activation to modulate A549 and HOP62 angiogenic potential. HUVECs plated on wells coated with Matrigel with the addition of A549 or HOP62 cell CM formed a characteristic capillary-like network in 3 days. On the other hand, when the cells were plated on Matrigel with the addition of CM of A549 or HOP62 treated with imiquimod, a lower number of tube structures were observed (Figure 1B,C). As control, HUVECs were plated in the presence of medium alone or medium with the addition of imiquimod (1 μg/mL) kept in the same condition of cell CM (37 °C for 18 h). Spontaneous tube structures were observed, without significant differences between the two conditions.

Since the formation of new sprout vessels implies endothelial cell migration and proliferation [27], we investigated the effects of CM of A549 with different expression levels of TLR7 on HUVEC and on human primary lung microvascular endothelial cell (HPLMEC) proliferation and migration. BrdU incorporation experiments demonstrated that CM of A549 cells silenced for TLR7 (two clones) induced S-phase entry of endothelial cells more efficiently than shCTR (a mass population) (Figure 1D). shTLR7 A549 (two clones) CM significantly increased HUVEC migration (Figure 1E) compared to shCTR CM. Consistently, CM of A549 cells treated with imiquimod induced HUVEC migration less efficiently than that of A549 left untreated (NT) (Figure 1E). Similar effects were detected with CM of HOP62 treated or untreated with imiquimod (Figure 1E).

These data demonstrate that TLR7 controls the release of soluble factors responsible for the angiogenic potential of lung cancer cells.

### 2.3. TLR7 Expression/Activation Status Correlates with the Expression of Angiogenic Mediators in NSCLC Cells

Since we demonstrated that TLR7 expression and activation status inversely correlated with the angiogenic potential of NSCLC cells, we investigated whether imiquimod or TLR7 silencing could modulate the expression of angiogenesis-related proteins in NSCLC cells. To this aim, we evaluated the expression of angiogenic proteins by using dedicated antibody arrays incubated with CM of A549 shTLR7 cells and of imiquimod-treated HOP62 cells or of the relative controls.

**Figure 2:**
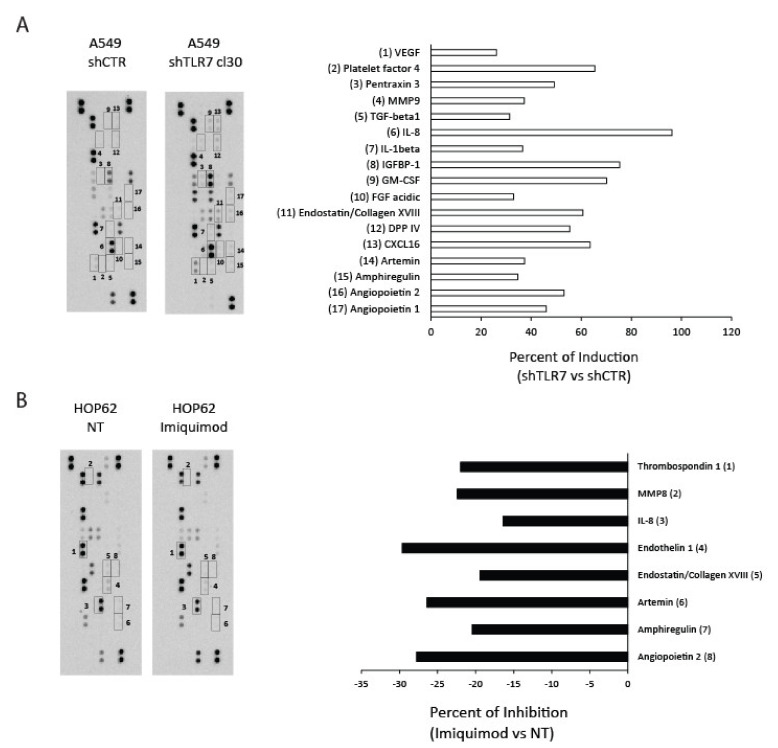
Effects of TLR7 modulation on NSCLC angiogenic potential. Analysis of proteins in conditioned media (CM) from A549 shCTR and shTLR7 clone 30 (**A**) or HOP62 treated or untreated with imiquimod (1 μg/mL) (**B**) using angiogenesis associated protein antibody arrays. The array images and the relative quantitative profiles of protein levels are shown.

The antibody arrays allowed us to detect 56.4% (31/55) of angiogenic proteins in A549 shCTR cells and 32.7% (18/55) in HOP62 cells (Figure 2). The mean of protein pixel density for each angiogenesis-related protein, normalized for the reference spots, was calculated and compared to the relative control. TLR7 silencing in A549 cells increased, compared to control, the levels of VEGF, platelet factor 4, pentraxin 3, MMP9, TGF-β, IL-8, IL-1β, IGFBP-1, GM-CSF, FGF acidic, endostatin/collagen XVIII, DPP IV, CXCL16, artemin, amphiregulin, angiopoietin 1 and 2 with changes greater than 30% (Figure 2A—A549 shTLR7 vs. shCTR). Furthermore, in HOP62 cells, TLR7 activation by imiquimod (1 μg/mL) downregulated, with changes superior than 15%, the levels of thrombospondin-1, MMP8, IL-8, endothelin 1, endostatin/collagen XVIII, artemin, amphiregulin, and angiopoietin 2 (Figure 2B—HOP62 imiquimod vs. NT). Results in line with that obtained in HOP62 cells were also obtained in A549 cells treated or untreated with imiquimod (Figure S3A).

In parallel, to evaluate whether TLR7 silencing could affect the tumorigenic potential of A549 cells, we xenografted shCTR (a mass population) and shTLR7 (two clones) A549 cells (5 × 10^6^ cells) in athymic mice and monitored tumor growth rate. At 3 weeks, no significant differences were observed between the two groups (Figure S3B). We hypothesize that a different and more physiological setting is needed in order to evaluate the impact of TLR7-mediated angiogenic modulation on tumor growth in vivo.

These data indicate that several angiogenic proteins are modulated by TLR7 activation/expression status in NSCLC cells.

### 2.4. TLR7-Silencing Decreases Pro-Resolving Potential and Increases Vascular Endothelial Growth Factor A (VEGF-A) Release of NSCLC Cells

To verify whether TLR7 silencing in NSCLC cells could modulate pro-resolving pathways, we correlated TLR7 expression to the levels of enzymes involved in pro-resolving mediator synthesis (i.e., ALOX5, ALOX15A, and ALOX15B), the release of pro-resolving mediators (i.e., RvD1 and LXB4), and of inflammatory autacoids (i.e., PGD2 and LTB4) [17,30], in parallel to the synthesis of vascular endothelial growth factor A (VEGF-A)—the main mediator of new vessel formation [21,27].

**Figure 3:**
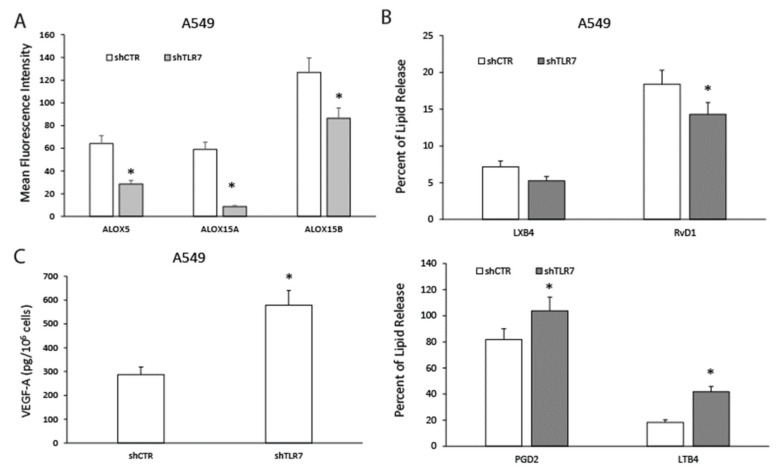
Effects of TLR7 silencing on the specialized pro-resolving mediator (SPM) biosynthetic machinery of NSCLC cells. (**A**) ALOX5, ALOX15A, and ALOX15B protein expression levels (mean fluorescence intensity), assessed by cytofluorimetric analysis, in A549 shTLR7 (two clones) cells and the relative control (shCTR, a mass population). Data are represented as mean ± SD of five independent experiments. * *p* < 0.05 compared to the relative control. (**B**) Pro-resolving and pro-inflammatory autacoids (LXB4, RvD1, PGD2, and LTB4) release in A549 shCTR (a mass population) or shTLR7 (two clones). Data are represented as mean ± SD of five independent experiments. * *p* < 0.05 compared to the relative control. (**C**) Spontaneous VEGF-A release in A549 shCTR (a mass population) or shTLR7 (two clones). Data are represented as mean ± SD of five independent experiments. * *p* < 0.05 compared to the control.

TLR7 knockdown significantly impaired ALOX5, ALOX15A, and ALOX15B protein expression in A549 cells as assessed by flow cytometric analysis (Figure 3A). Similar data were obtained when ALOX5, ALOX15A, and ALOX15B mRNAs were evaluated (Figure S4A).

A549 shTLR7 clones displayed significantly reduced release of RvD1 but not of LXB4—evaluated as examples of SPMs—and increased levels of PGD2 and LTB4, examples of pro-inflammatory autacoids (Figure 3B) compared to controls. The inability of TLR7 to modulate LXB4 release is in line with the observation made, by lipidomic profiling, in mice lung following TLR7 activation or silencing [24].

Consistently with data obtained with the antibody array, we demonstrated that shTLR7 A549 cells expressed increased levels of VEGF-D and Ang-1 mRNAs (Figure S4B). Furthermore, shTLR7 clones released significantly higher levels of VEGF-A in A549 cell culture supernatants (Figure 3C) compared to control.

These data confirm that TLR7 expression is directly linked to pro-resolving pathway components’ expression and inversely correlated to angiogenic mediator production.

### 2.5. TLR7 Activation Sustains Pro-Resolving Pathway Components’ Expression and Inhibits Angiogenic Mediators’ Production in NSCLC Cells

To study whether not only TLR7 expression levels but also TLR7 activation could modulate pro-resolving pathways in NSCLC cells and whether this could mediate an anti-angiogenic response, we treated A549 and HOP62 cells with imiquimod (1 μg/mL), agonist to TLR7, and as a positive control, with the SPMs RvD1 (1 nM) or LXB4 (1 nM) for 3 h to evaluate, by qPCR, the expression levels of enzymes responsible for pro-resolving mediator synthesis (ALOX5, ALOX15A, ALOX15B) of receptors recognizing pro-resolving mediators (GPR32, ChemR23) and of pro-angiogenic mediators (VEGF-A, -B, -C, -D, Ang1, CXCL1).

**Figure 4:**
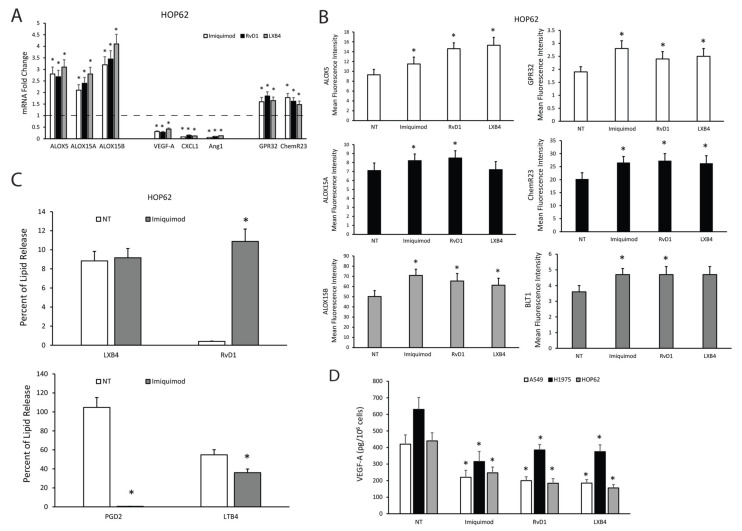
Effects of TLR7 activation on SPM biosynthetic machinery and angiogenic potential of NSCLC cells. (**A**) ALOX5, ALOX15A, ALOX15B, VEGF-A, CXCL1, Ang1, GPR32, and ChemR23 mRNA fold change in HOP62 cells treated with imiquimod (1 μg/mL), RvD1 (1 nM), or LXB4 (1 nM) for 3 h. Data are represented as mean ± SD of five independent experiments. * *p* < 0.05 compared to the control (dotted line). (**B**) ALOX5, ALOX15A, ALOX15B, GPR32, ChemR23, and BLT1 protein expression levels (mean fluorescence intensity), assessed by cytofluorimetric analysis, in HOP62 cells treated with imiquimod (1μg/mL), RvD1 (1 nM), or LXB4 (1 nM) for 6 h. Data are represented as mean ± SD of three independent experiments. * *p* < 0.05 compared to the control (NT). (**C**) Pro-resolving and pro-inflammatory autacoids (LXB4, RvD1, PGD2, and LTB4) release in HOP62 cells treated or untreated with imiquimod (1 μg/mL, 18 h). Data are represented as mean ± SD of five independent experiments. * *p* < 0.05 compared to the control. (**D**) VEGF-A release in A549, H1975, and HOP62 cells treated with imiquimod (1 μg/mL), RvD1 (1 nM), or LXB4 (1 nM) for 10 h. Data are represented as mean ± SD of five independent experiments. * *p* < 0.05 compared to the control.

Figure 4A shows that imiquimod was able to significantly increase pro-resolving pathway components and significantly reduce angiogenic mediators’ mRNA expression similar to RvD1 and LXB4 in HOP62 cells. Similar results were obtained in A549 and H1975 cells (Figure S5A). To confirm these data, we also evaluated the protein expression levels of ALOX5, ALOX15A, ALOX15B, GPR32, ChemR23, and BLT1 in HOP62 cells treated for 6 h with an optimal concentration of imiquimod (1 μg/mL), RvD1 (1 nM), and LXB4 (1 nM) by FACS analysis. Our results demonstrated that imiquimod, RvD1, and LXB4 were able to significantly increase enzyme and receptor expression in HOP62 cells (Figure 4B and Figure S5B). Similar results were obtained in A549 and H1975 cells (Figure S5C).

To corroborate our hypothesis, we evaluated the release of the SPMs RvD1 and LXB4 and of the pro-inflammatory autacoids PGD2 and LTB4 in HOP62 cells following imiquimod (1 μg/mL) treatment for 18 h. No changes in the levels of LXB4 were observed in treated cells compared to control (Figure 4C), consistently with the data available in the literature [24] and obtained by us on silenced clones. However, imiquimod significantly increased the release of RvD1 (Figure 4C), and significantly reduced PGD2 and LTB4 release (Figure 4C) from HOP62 cells.

Finally, we measured the VEGF-A content in supernatants of A549, H1975, and HOP62 cells treated or untreated for 10 h with imiquimod (1 μg/mL), RvD1 (1 nM), and LXB4 (1 nM). Imiquimod, similarly to RvD1 and LXB4, was able to significantly reduce VEGF-A release from the three NSCLC cells used (Figure 4D).

Our data support the hypothesis that TLR7 is the main PRR responsible for the modulation of pro-resolving and anti-angiogenic responses in NSCLC.

### 2.6. SPMs Inhibit the Production of Angiogenic Mediators and Restore the Expression of Pro-Resolving Pathway Components in NSCLC Cells

To determine whether the increased angiogenic potential of NSCLC cells depleted of TLR7 could be due to their defective pro-resolving pathways’ expression, we assessed the effects of exogenously added SPMs (RvD1 and LXB4) on pro-angiogenic activity of NSCLC cells silenced for TLR7.

**Figure 5:**
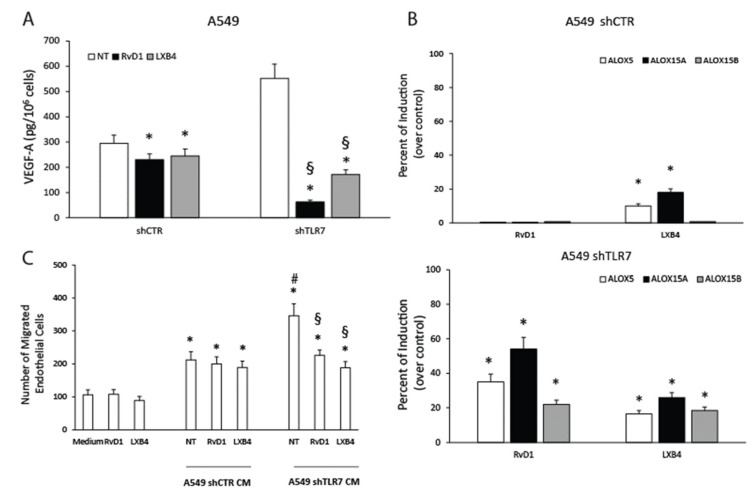
Anti-angiogenic and pro-resolving effects of SPMs in NSCLC cells. (**A**) VEGF-A release in A549 shCTR (a mass population) and shTLR7 (two clones) (10 h) treated or untreated (NT) with RvD1 (1 nM) or LXB4 (1 nM). Data are represented as mean ± SD of five independent experiments. * *p* < 0.05 compared to the control. (**B**) Percent of induction of ALOX5, ALOX15A, and ALOX15B protein expression levels (mean fluorescence intensity), assessed by cytofluorimetric analysis, in A549 shCTR (a mass population) and A549 shTLR7 (two clones) cells following RvD1 (1 nM) or LXB4 (1 nM) treatment (6 h). Data are represented as mean ± SD of five independent experiments. * *p* < 0.05 compared to the control. (**C**) Number of HUVEC migrated cells toward CM from A549 shCTR and shTLR7 treated or untreated with RvD1 (1 nM) or LXB4 (1 nM). The effects of the addition of the two SPMs to the medium (DMEM 1% FBS) is also shown. Data are represented as mean ± SD of three independent determinations. * *p* < 0.05 compared to the relative control; # *p* < 0.05 compared to shCTR; § *p* < 0.05 compared to the relative NT.

To this aim, we treated or left untreated A549 shTLR7 and shCTR cells with RvD1 (1 nM) or LXB4 (1 nM) and evaluated the levels of pro-angiogenic mediators both at mRNA and protein levels. RvD1 and LXB4 suppressed VEGF-D mRNA expression in shTLR7 cells but not in shCTR cells (Figure S6A). Ang1 mRNA levels were significantly reduced by SPM treatment in shTLR7 cells and, to a lesser extent, also in A549 shCTR cells (Figure S6A). As expected, we demonstrated that both RvD1 and LXB4 were able to significantly reduce VEGF-A release in both A549 shTLR7 (≈70% of inhibition) and shCTR cells (≈20% of inhibition) (Figure 5A).

Interestingly, RvD1 and LXB4 treatment significantly increased the expression of ALOX5, ALOX15A, ALOX15B at mRNA (Figure S6B), and protein levels (Figure 5B and Figure S6C), thereby triggering a positive feed-forward loop. The responses of A549 shCTR to RvD1 and LXB4 were significantly less efficient than those observed in A549 shTLR7 cells (Figure 5B and Figure S6C). Similar data were also obtained by evaluating the effects of RvD1 and LXB4 on the mRNA expression of the SPM receptors (GPR32 and ChemR23) (Figure S6B).

Consistent with a significant reduction in VEGF-A release, the functional angiogenic potential of shTLR7 NSCLC cells was also significantly affected by SPM addition as evaluated by an endothelial cell migration assay (Figure 5C).

These data demonstrate that the increased angiogenic potential of NSCLC cells silenced for TLR7 is due to the reduced pro-resolving potential as it can be restored by adding back SPMs.

### 2.7. TLR7-Mediated Pro-Resolving Response in NSCLC Cells Requires MAPK and STAT3 Activation

We then asked which signaling pathways were involved in the TRL7-mediated pro-resolving and anti-angiogenic response of NSCLC cells. To this aim, we evaluated which pathways could be modulated upon imiquimod treatment or TLR7 silencing in NSCLC cells.

**Figure 6:**
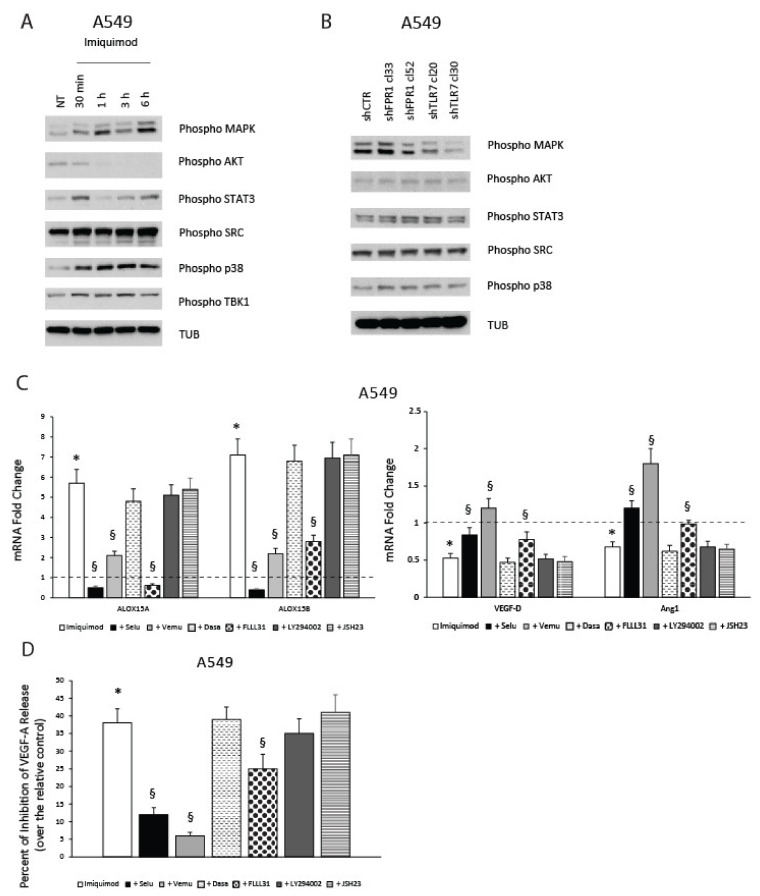
Signaling pathways involved in anti-angiogenic and pro-resolving functions of TLR7. (**A**) Activation kinetics of MAPK, AKT, STAT3, SRC, p38, and TKB1 in A549 cells treated or not for the indicated time points with imiquimod (1 μg/mL), assessed by Western blot for their phosphorylated forms. α-tubulin (TUB) was used as a normalization marker. A representative experiment is shown. (**B**) Constitutive MAPK, AKT, STAT3, SRC, and p38 activation levels in A549 shCTR (a mass population), shFPR1 (clones 33 and 52), and shTLR7 (clones 20 and 30) assessed by Western blot for their phosphorylated forms. α-tubulin (TUB) was used as a normalization marker. A representative experiment is shown. (**C**) Effects of selumetinib (Selu)—MEK inhibitor 10 μM, vemurafenib (Vemu)—BRAF inhibitor 10 μM, dasatinib (Dasa)—SRC inhibitor 1 μM, FLLL31—STAT3 inhibitor 10 μM, LY294002—AKT inhibitor 15 μM, and JSH23—NF-kB inhibitor 5 μM on imiquimod-induced modulation of ALOX15A, ALOX15B, VEGF-D, and Ang1 mRNA expression. Data are represented as mean ± SD of three independent experiments. * *p* < 0.05 compared to untreated cells (dotted line). § *p* < 0.05 compared to imiquimod-treated cells. (**D**) Effects of selumetinib—MEK inhibitor 10 μM, vemurafenib—BRAF inhibitor 10 μM, dasatinib—SRC inhibitor 1 μM, FLLL31—STAT3 inhibitor 10 μM, LY294002—AKT inhibitor 15 μM, and JSH23—NF-kB inhibitor 5 μM on imiquimod-induced VEGF-A release inhibition. Data are represented as mean ± SD of three independent experiments. * *p* < 0.05 compared to untreated cells. § *p* < 0.05 compared to imiquimod-treated cells.

We found that MAPK, STAT3, SRC, p38 and, as positive control, TBK1 signaling pathways [4] were induced in A549 cells treated with imiquimod (1 μg/mL) for different times (30 min–6 h) (Figure 6A). No activation of AKT was observed in A549 cells treated with imiquimod (Figure 6A). Similar results were obtained in HOP62 cells (Figure S7A). No detectable levels of NF-kB, assessed through phospho-IKKα/β and phospho-p65 antibodies, were found in A549 cells. Then, we compared the basal levels of MAPK, AKT, STAT3, SRC, and p38 activation in two shFPR1, two shTLR7 clones, and an A549 shCTR mass population. As shown in Figure 6B, no significant differences in the activation levels were observed in A549 shFPR1, shTLR7, and shCTR cells for AKT, STAT3, SRC, and p38 activation levels. Interestingly, the activation levels of MAPK were lower in A549 shTLR7 cells compared to shFPR1 and shCTR cells. These last results suggest the presence of an endogenous ligand to TLR7 in our cells, as already demonstrated in other systems [31]. Intensity ratio for the data presented in Figure 6 and for two other experiments are shown in Figure S7B. To complete the comprehension of signaling pathways involved in the anti-angiogenic effect of TLR7, an evaluation of MAPK and STAT3 signaling activation in A549 cells upon treatment with RvD1 (1nM) or LXB4 (1 nM) is also shown (Figure S7C).

Consistent with the activation of MAPK signaling in A549 cells following imiquimod treatment and with its reduced basal level in shTLR7 cells compared to controls, specific inhibitors of this pathway (selumetinib—MEK inhibitor 10 μM; vemurafenib—BRAF inhibitor 10μM) [32,33] were able to significantly revert ALOX15A and ALOX15B mRNA induction sustained by imiquimod treatment (3 h) (Figure 6C). Similarly, imiquimod-mediated reduction of VEGF-D and Ang1 was reverted by pre-incubation of cells with selumetinib and vemurafenib (Figure 6C). No effects of dasatinib (1 μM)—inhibitor of SRC [34], LY294002 (15 μM)—inhibitor of AKT [21], and JSH 23 (5 μM)—inhibitor of NF-kB [35] were detected on imiquimod effects on pro-resolving or angiogenic mediators’ synthesis (Figure 6C).

Although no differences in the basal activation levels of STAT3 were detected in A549 shCTR, shFPR1, and shTLR7 cells, the activation of STAT3 mediated by imiquimod (Figure 6A,B) was relevant in the induction of pro-resolving pathways components and in the inhibition of angiogenic mediators, as demonstrated by the FLLL31 (10 μM)-mediated inhibition of STAT3 [36] (Figure 6C). The key effects of MAPK and STAT3 activation on TLR7-mediated inhibition of angiogenesis was also confirmed through the measurement of VEGF-A release from A549 cells (Figure 6D).

These data suggest that MAPK and STAT3 signaling are significantly involved in pro-resolving and anti-angiogenic response of NSCLC cells to imiquimod.

## 3. Discussion

Lung cancer is the most common cancer worldwide and the main cause of cancer-associated mortality [37]. A crucial role of angiogenesis in NSCLC progression is supported by the association between elevated intratumor VEGF levels and a poorer prognosis or a more aggressive disease in NSCLC patients and by the evidence that bevacizumab, an anti-VEGF-A antibody, is currently used for the treatment of this type of cancer, showing improvement in patients’ survival [38]. Although a benefit has been demonstrated from the use of bevacizumab in several tumor types, the approach of targeting only VEGF-A with bevacizumab could be responsible for resistance phenomena via activation of alternative angiogenic pathways [39,40]. Thus, an approach affecting more than one pro-angiogenic mediator may target more effectively angiogenesis in lung cancer and other tumor types.

We recently demonstrated that the innate immune receptor FPR1 is crucial in gastric cancer (GC) to sustain the constitutive production of pro-resolving mediators that restrains tumor growth by suppressing angiogenesis [17,21]. We demonstrated that FPRs are important in the physiologic wound repair of the gastrointestinal epithelium [6,7,41] and that in GC its ablation/pharmacological inhibition increased the tumorigenic potential causing a drop in the endogenous levels of pro-resolving pathway components and a concomitant increase in the angiogenic potential of GC cells.

We hypothesized that similar mechanisms could intervene in all tissues exposed to the external environment that should maintain a higher pro-resolving tone, compared to other districts being constitutively exposed to pro-inflammatory stimuli. In particular, different classes of innate immune receptors could play a key homeostatic role in different tissues depending on the most common PAMPs or DAMPs present in the specific district.

**Figure 7:**
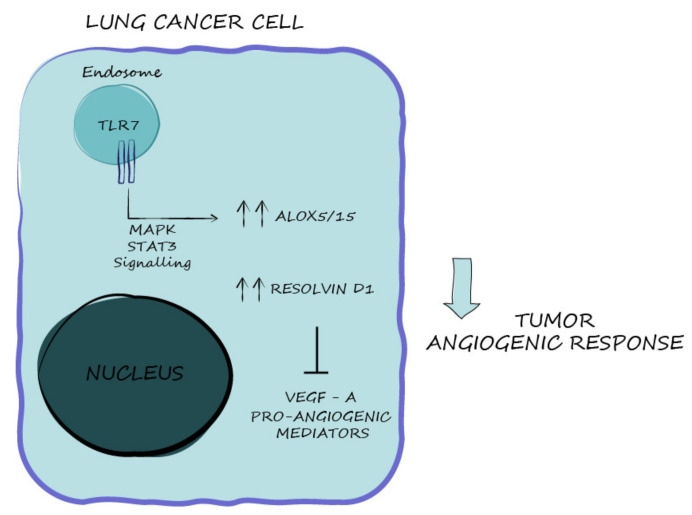
Schematic representation of mechanisms involved in TLR7-mediated angiogenic inhibition.

Koltsida et al. demonstrated that TLR7 is critical for the resolution of airway inflammation due to its ability to control the mobilization of the ω-3 docosahexaenoic acid (DHA) biosynthetic pathway and the generation of D-series SPMs [24]. TLR7 and its closely related receptor, TLR8, play an important role in the immune response to viral infection; they recognize single-stranded RNAs as natural ligand but have also been demonstrated to sense imidazoquinolines and nucleoside analogues [42]. We hypothesized that TLR7 is the main sensor of damages and modulator of the balance between inflammatory and pro-resolving responses in the context of lung cancer. Consistent with this hypothesis, we demonstrated that TLR7 expression/activation directly correlated with pro-resolving pathway components’ expression, which in turn inhibits NSCLC angiogenic potential (Figure 7).

Several other reports sustain an antitumor effect of pro-resolving pathways in NSCLC. It has been demonstrated that SPMs exert anticancer effects in NSCLC experimental models by inhibiting epithelial-to-mesenchymal transition [43]. Moreover, chemotherapy-induced cell debris can stimulate lung cancer progression, but this effect can be inhibited by SPMs. Indeed, SPMs potentiate macrophage efferocytosis by enhancing debris clearance and counteract inflammatory cytokine activity [44]. SPM precursors, ω-6 and ω-3 PUFAs, decrease lung-cancer cell growth by inducing cell death [45]. Furthermore, ALOX15 is considered a tumor suppressor, since its expression or activity is frequently decreased during lung carcinogenesis [46,47].

TLR7 role in cancer is still debated. TLR7 signaling can favor anti-apoptotic, pro-survival, and chemoresistance activities of NSCLC through the activation of the NF-kB pathway [48,49]. Consistently, the administration of the TLR7 agonist loxoribine to mice grafted with lung adenocarcinoma cells increased chemotherapeutic resistance. Of note, no effects on xenograft growth were observed following TLR7 silencing in NSCLC cells [49], as also confirmed by us. In a different experimental setting, by using a syngeneic Lewis lung carcinoma (LLC) cell system in C57BL/6 mice, Dr. Cremer’s group was able to demonstrate that the pro-tumorigenic role of TLR7 depends on its expression on cancer cells and on the recruitment of myeloid-derived suppressor cells (MDSCs) infiltrating the tumor [50]. Moreover, tumor cell TLR7 expression inversely associated with patients’ survival [49]. On the other hand, the association of TLR7 expression with better clinical outcome comes from studies considering the entire tumor microenvironment: higher mRNA expression of TLR7 in NSCLC samples significantly associates with overall survival in NSCLC patients as published by Bauer et al. and also found by us [51]. This evidence suggests that the TLR7 functions in cancer progression are complex and might be dependent on the tumor context.

The Cremer group also observed that, despite the activation of NF-kB by TLR7, some pro-inflammatory and angiogenic mediators (IL-1, IL-7, IL-15, IL-12, AGPT4, PDGFβ, AGPT1L) are suppressed in NSCLC cells. In support of our observations, they hypothesized a dual role of TLR7 as a function of the different signaling pathways activated [48]. Thus, the pro-tumorigenic role of TLR7 in NSCLC cells depends on NF-kB; by contrast, we showed that the pro-resolving and anti-angiogenic functions mediated by TLR7 depend on MAPK and STAT3 pathway activation (Figure 7). The role of the MAPK signaling cascade activated by TLRs in immune induction, inflammation, and modulation of angiogenic response remains to be defined and could be context dependent [52]. In any case, a specific role of the MAPK signaling in mediating both pro-inflammatory and anti-inflammatory processes has been recognized, as several molecules targeting this pathway often cause an inhibition of the negative feedback controlling inflammation [53]. Moreover, the role of STAT3 as a tumor-suppressor is controversial: several studies point to a pro-angiogenic role of STAT3 in several cell contexts [54]; but STAT3 also shows tumor suppressor function in different cancer contexts by activating various molecular mechanisms [55,56]. It is possible that STAT3 signaling could be activated downstream of the MAPK pathways in response to TLR7 activation, as already demonstrated in other contexts [57], and that it could be the main transcription factor responsible for the pro-resolving/anti-angiogenic response of NSCLC cells as already reported in GC [17].

Due to their potent immuno-stimulatory activity on immune cells, ligands to TLR7 have been evaluated as immunotherapeutic agents with anticancer activity in several pre-clinical models [58]. A very recent study highlights the strong antitumor activity of resiquimod (TLR7 agonist, analogue to imiquimod) in NSCLC due to its effects of immuno-modulation of the tumor microenvironment. In particular, the authors demonstrate that the TLR7 agonist had a superior tumor inhibitory effect in a metastatic model of lung adenocarcinoma, relative to anti-PD1 therapy or platinum-based chemotherapy [59]. Our data identifying TLR7 as a modulator of the angiogenic response of NSCLC could enrich and complete the scenario of the effects of TLR7 agonists in NSCLC cancer. These results describing the antitumor effects of TLR7 have been obtained using imidazoquinolines (i.e., imiquimod and resiquimod) as TLR7 agonists. Instead, the pro-tumorigenic role of TLR7 has been demonstrated by using nucleoside analogues (i.e., loxoribine and CL264) to activate TLR7 [49,50]. The possibility that diverse agonists to TLR7 could exert different functions by also preferentially activating MAPK/STAT3 or NF-kB pathways cannot be excluded.

Our studies unveil new biology with the identification of molecular mechanisms linking lipid metabolism and inflammation resolution with gastric and non-small cell lung cancer, opening the possibility of innovative therapeutic strategies for the clinical management of these types of neoplasia.

## 4. Materials and Methods

### 4.1. Reagents

Imiquimod, LY294002, and FLLL31 were from Sigma Aldrich (St. Louis, MO, USA); RvD1 and LXB4 from Cayman Chemical (Ann Arbor, MI, USA); selumetinib, vemurafenib, and dasatinib were from Selleckchem (Houston, TX, USA).

### 4.2. Cell Culture

A549, NCI-H1975 (H1975 throughout the text), and HOP62 lung cancer cells were grown as previously described [60,61,62]. Human primary lung microvascular endothelial cells (HPLMECs) and human umbilical vein endothelial cells (HUVECs) from Cell Biologics were grown in human endothelial cell medium with the addition of VEGF, heparin, EGF, FGF, hydrocortisone, L-glutamine, antibiotic–antimycotic Solution, and FBS according to the manufacturer’s instructions (Cell Biologics, Chicago, IL, USA). To generate A549 cells stably expressing FPR1 or TLR7 shRNA, we used pools of five constructs (shRNA FPR1-Qiagen, Valencia, CA, USA; shRNA TLR7-Origene, Rockville, MD, USA) containing 21-mer short hairpin RNAs (shRNA) directed to various coding regions of each target gene. Conditioned media from NSCLC cells (CM) were collected 18 h after incubation in 1% serum containing media or cell activation with specific stimuli.

### 4.3. Tubule Formation

The formation of network-like structures by HUVECs (Cell Biologics) on an extracellular matrix (ECM)-like 3D gel consisting of Matrigel^®^ (BD Biosciences, Mississauga, ON, Canada) was performed as previously described and validated [63]. HUVECs (5 × 10^4^) were seeded on a Matrigel matrix in the presence of cell culture supernatants. After incubation, HUVECs underwent differentiation into capillary-like tube structures. Tubule formation was defined as a structure exhibiting a length four times its width. Network formation was observed using an inverted-phase contrast microscope (Zeiss, Oberkochen, Germany). Representative fields were taken [63] and the number of branching points counted in five fields was presented as mean ± SD of three experiments. 

### 4.4. S-Phase Entry

S-phase entry was evaluated by BrdU incorporation and flow cytometric analysis. Endothelial cells were grown, serum-deprived, and treated with the indicated cell culture supernatants for 36 h. BrdU was added at a concentration of 10 μM for the last 2 h. BrdU-positive cells were revealed with Texas-Red-conjugated secondary antibodies (Abs) (Jackson ImmunoResearch Laboratories, West Grove, PA, USA). Fluorescence was analyzed by a FACSCalibur cytofluorimeter using CellQuest software (BD Biosciences) [64].

### 4.5. Endothelial Cell Migration

Chemotaxis was elicited using a Boyden chamber assay. We used a 48-well microchemotaxis chamber (NeuroProbe, Gaithersburg, MD, USA) and 8-μm-pore polycarbonate membranes (Nucleopore, Pleasanton, CA, USA) coated with 10 μg/mL fibronectin (Sigma-Aldrich) as described elsewhere [65].

### 4.6. Protein Array

The expression of angiogenesis-related proteins in NSCLC cell CM was determined using the Human Angiogenesis Array Kit (R&D Systems, Minneapolis, MI, USA) according to the manufacturer’s instructions. The data from developed X-ray films were digitalized and quantified using Image J analysis software [66].

### 4.7. Flow Cytometry

Cells were incubated (30 min at 4 °C) with specific or isotype control antibodies (Abs). Anti-ALOX5, ALOX15A, and ALOX15B Abs were from Santa Cruz Biotechnology (Dallas, TX, USA), anti-GPR32 was from Acris (Herford, Germany), anti-BLT1 from LSBio (Seattle, WA, USA), anti-ChemR23 and anti-FPR1 from R&D systems (Minneapolis, MI, USA), anti-TLR7 from Invitrogen (Carlsbad, CA, USA). Cells were analyzed with a FACSCalibur cytofluorimeter using CellQuest software (BD Biosciences). When necessary, we performed cell membrane permeabilization using the Cytofix/Cytoperm kit (BD Biosciences).

### 4.8. ELISA and EIA Assays

VEGF-A contents in culture supernatants were measured with a commercially available ELISA (R&D Systems). RvD1, LTB4, PGE2, and LXB4 contents in culture supernatants were measured with a commercially available EIA (Cayman Chemical, Ann Arbor, MI, USA) [17].

### 4.9. Real-Time PCR

Total RNA was isolated and retro-transcribed as previously described. Real-time quantitative PCR was performed as reported elsewhere [67]. Normalization was performed using β-actin mRNA levels. Primer sequences are listed in Table S1.

### 4.10. Protein Studies

Protein extractions and immunoblotting experiments were performed according to standard procedures [68]. Anti-phospho MAPK, -phospho AKT, -phospho STAT3, -phospho SRC, -phospho p38, and -phospho TKB1 antibodies for Western blot analysis were from Cell Signaling Technology (Beverly, MA, USA). Monoclonal anti-tubulin antibody was from Sigma-Aldrich. Secondary anti-mouse and anti-rabbit antibodies were coupled to horseradish peroxidase (Bio-Rad, Hercules, CA, USA).

### 4.11. Statistical Analysis

Values from groups were compared by using the paired Student t-test or the Duncan test. A *p*-value <0.05 was considered statistically significant. Clinico-pathologic parameters in relation to TLR7 expression were plotted using the cBioPortal. Co-expression data were obtained according to the cBioPortal online instructions: a log–rank test was provided to identify the significance of the Spearman’s correlation coefficient between the mRNA expression z-Scores (RNASeq V2 RSEM).

## 5. Conclusions

To date, there are only a small number of studies investigating the correlation and the mechanisms linking pro-resolving pathways and cancer in humans. The discovery that innate immune receptors (i.e., FPR1 in gastric cancer and TLR7 in lung cancer) might control PUFA metabolism, inflammation resolution, angiogenesis, and cancer opens new possibilities to be exploited for cancer treatment. The administration of innate immune agonists, modulators of pro-resolving pathways, or increasing ω-3 or ω-6 diet consumption might represent novel therapeutic approaches for the treatment/prevention of cancers. Furthermore, components of pro-resolving pathways may be used as novel risk factors or prognostic markers for these types of cancers.

## Data Availability

The study utilized publically available datasets from cBioPortal for Cancer Genomics database (http://www.cbioportal.org (accessed on 4 January 2021)).

## Supplementary Materials

The following are available online at https://www.mdpi.com/2072-6694/13/4/740/s1, Supplementary Methods; Figure S1: TLR7 correlation to NSCLC patients’ characteristics; Figure S2: FPR1 and TLR7 expression in NSCLC cells; Figure S3: Effects of TLR7 modulation on NSCLC angiogenic and tumorigenic potential; Figure S4: Effects of TLR7 silencing on SPM biosynthesis machinery and angiogenic potential of NSCLC cells; Figure S5: Effects of TLR7 activation on SPM biosynthetic machinery and angiogenic potential of NSCLC cells; Figure S6: Anti-angiogenic and pro-resolving effects of SPMs in NSCLC cells; Figure S7: Signaling pathways involved in anti-angiogenic and pro-resolving functions of TLR7, Table S1: List of primers.

## References

[1] Thompson M.R., Kaminski J.J., Kurt-Jones E.A., Fitzgerald K.A. (2011). Pattern recognition receptors and the innate immune response to viral infection. Viruses.

[2] Takeuchi O., Akira S. (2010). Pattern recognition receptors and inflammation. Cell.

[3] Prevete N., Liotti F., Marone G., Melillo R.M., de Paulis A. (2015). Formyl peptide receptors at the interface of inflammation, angiogenesis and tumor growth. Pharmacol. Res..

[4] Kawasaki T., Kawai T. (2014). Toll-like receptor signaling pathways. Front. Immunol..

[5] Fukata M., Arditi M. (2013). The role of pattern recognition receptors in intestinal inflammation. Mucosal Immunol..

[6] Prevete N., de Paulis A., Sgambato D., Melillo R.M., D’Argenio G., Romano L., Zagari R.M., Romano M. (2018). Role of Formyl Peptide Receptors in Gastrointestinal Healing. Curr. Pharm. Des..

[7] Gravina A.G., Prevete N., Tuccillo C., De Musis C., Romano L., Federico A., de Paulis A., D’Argenio G., Romano M. (2018). Peptide Hp(2–20) accelerates healing of TNBS-induced colitis in the rat. United Eur. Gastroenterol. J..

[8] Hacker H., Redecke V., Blagoev B., Kratchmarova I., Hsu L.C., Wang G.G., Kamps M.P., Raz E., Wagner H., Hacker G. (2006). Specificity in Toll-like receptor signalling through distinct effector functions of TRAF3 and TRAF6. Nature.

[9] Dufton N., Hannon R., Brancaleone V., Dalli J., Patel H.B., Gray M., D’Acquisto F., Buckingham J.C., Perretti M., Flower R.J. (2010). Anti-inflammatory role of the murine formyl-peptide receptor 2: Ligand-specific effects on leukocyte responses and experimental inflammation. J. Immunol..

[10] Prevete N., Liotti F., Amoresano A., Pucci P., de Paulis A., Melillo R.M. (2018). New perspectives in cancer: Modulation of lipid metabolism and inflammation resolution. Pharmacol. Res..

[11] Serhan C.N., Chiang N., Van Dyke T.E. (2008). Resolving inflammation: Dual anti-inflammatory and pro-resolution lipid mediators. Nat. Rev. Immunol..

[12] Serhan C.N. (2014). Pro-Resolving Lipid Mediators Are Leads for Resolution Physiology. Nature.

[13] Gronert K., Maheshwari N., Khan N., Hassan I.R., Dunn M., Laniado Schwartzman M. (2005). A Role for the Mouse 12/15-Lipoxygenase Pathway in Promoting Epithelial Wound Healing and Host Defense. J. Biol. Chem..

[14] Tang Y., Zhang M.J., Hellmann J., Kosuri M., Bhatnagar A., Spite M. (2013). Proresolution therapy for the treatment of delayed healing of diabetic wounds. Diabetes.

[15] Miyata J., Fukunaga K., Iwamoto R., Isobe Y., Niimi K., Takamiya R., Takihara T., Tomomatsu K., Suzuki Y., Oguma T. (2013). Dysregulated synthesis of protectin D1 in eosinophils from patients with severe asthma. J. Allergy Clin. Immunol..

[16] Leedom A.J., Sullivan A.B., Dong B., Lau D., Gronert K. (2010). Endogenous LXA4 circuits are determinants of pathological angiogenesis in response to chronic injury. Am. J. Pathol..

[17] Prevete N., Liotti F., Illiano A., Amoresano A., Pucci P., de Paulis A., Melillo R.M. (2017). Formyl peptide receptor 1 suppresses gastric cancer angiogenesis and growth by exploiting inflammation resolution pathways. Oncoimmunology.

[18] Gilligan M.M., Gartung A., Sulciner M.L., Norris P.C., Sukhatme V.P., Bielenberg D.R., Huang S., Kieran M.W., Serhan C.N., Panigrahy D. (2019). Aspirin-triggered proresolving mediators stimulate resolution in cancer. Proc. Natl. Acad. Sci. USA.

[19] Sulciner M.L., Gartung A., Gilligan M.M., Serhan C.N., Panigrahy D. (2018). Targeting lipid mediators in cancer biology. Cancer Metastasis Rev..

[20] Zeromski J., Kaczmarek M., Boruczkowski M., Kierepa A., Kowala-Piaskowska A., Mozer-Lisewska I. (2019). Significance and Role of Pattern Recognition Receptors in Malignancy. Arch. Immunol. Ther. Exp..

[21] Prevete N., Liotti F., Visciano C., Marone G., Melillo R.M., de Paulis A. (2015). The formyl peptide receptor 1 exerts a tumor suppressor function in human gastric cancer by inhibiting angiogenesis. Oncogene.

[22] Otani T., Ikeda S., Lwin H., Arai T., Muramatsu M., Sawabe M. (2011). Polymorphisms of the formylpeptide receptor gene (FPR1) and susceptibility to stomach cancer in 1531 consecutive autopsy cases. Biochem. Biophys. Res. Commun..

[23] Seifert R., Wenzel-Seifert K. (2001). Defective Gi protein coupling in two formyl peptide receptor mutants associated with localized juvenile periodontitis. J. Biol. Chem..

[24] Koltsida O., Karamnov S., Pyrillou K., Vickery T., Chairakaki A.D., Tamvakopoulos C., Sideras P., Serhan C.N., Andreakos E. (2013). Toll-like receptor 7 stimulates production of specialized pro-resolving lipid mediators and promotes resolution of airway inflammation. EMBO Mol. Med..

[25] Gao J., Aksoy B.A., Dogrusoz U., Dresdner G., Gross B., Sumer S.O., Sun Y., Jacobsen A., Sinha R., Larsson E. (2013). Integrative analysis of complex cancer genomics and clinical profiles using the cBioPortal. Sci. Signal..

[26] Cerami E., Gao J., Dogrusoz U., Gross B.E., Sumer S.O., Aksoy B.A., Jacobsen A., Byrne C.J., Heuer M.L., Larsson E. (2012). The cBio cancer genomics portal: An open platform for exploring multidimensional cancer genomics data. Cancer Discov..

[27] Senger D.R., Davis G.E. (2011). Angiogenesis. Cold Spring Harb. Perspect. Biol..

[28] Crozat K., Beutler B. (2004). TLR7: A new sensor of viral infection. Proc. Natl. Acad. Sci. USA.

[29] Javaid N., Choi S. (2020). Toll-like Receptors from the Perspective of Cancer Treatment. Cancers.

[30] Serhan C.N., Chiang N., Dalli J. (2015). The resolution code of acute inflammation: Novel pro-resolving lipid mediators in resolution. Semin. Immunol..

[31] Negishi H., Endo N., Nakajima Y., Nishiyama T., Tabunoki Y., Nishio J., Koshiba R., Matsuda A., Matsuki K., Okamura T. (2019). Identification of U11snRNA as an endogenous agonist of TLR7-mediated immune pathogenesis. Proc. Natl. Acad. Sci. USA.

[32] Xing J., Liu R., Xing M., Trink B. (2011). The BRAFT1799A mutation confers sensitivity of thyroid cancer cells to the BRAFV600E inhibitor PLX4032 (RG7204). Biochem. Biophys. Res. Commun..

[33] Ball D.W., Jin N., Rosen D.M., Dackiw A., Sidransky D., Xing M., Nelkin B.D. (2007). Selective growth inhibition in BRAF mutant thyroid cancer by the mitogen-activated protein kinase kinase 1/2 inhibitor AZD6244. J. Clin. Endocrinol. Metab..

[34] Lombardo L.J., Lee F.Y., Chen P., Norris D., Barrish J.C., Behnia K., Castaneda S., Cornelius L.A., Das J., Doweyko A.M. (2004). Discovery of N-(2-chloro-6-methyl-phenyl)-2-(6-(4-(2-hydroxyethyl)-piperazin-1-yl)-2-methylpyrimidin-4-ylamino)thiazole-5-carboxamide (BMS-354825), a dual Src/Abl kinase inhibitor with potent antitumor activity in preclinical assays. J. Med. Chem..

[35] Shin H.M., Kim M.H., Kim B.H., Jung S.H., Kim Y.S., Park H.J., Hong J.T., Min K.R., Kim Y. (2004). Inhibitory action of novel aromatic diamine compound on lipopolysaccharide-induced nuclear translocation of NF-kappaB without affecting IkappaB degradation. FEBS Lett..

[36] Lin L., Hutzen B., Zuo M., Ball S., Deangelis S., Foust E., Pandit B., Ihnat M.A., Shenoy S.S., Kulp S. (2010). Novel STAT3 phosphorylation inhibitors exhibit potent growth-suppressive activity in pancreatic and breast cancer cells. Cancer Res..

[37] Visconti R., Morra F., Guggino G., Celetti A. (2017). The between Now and Then of Lung Cancer Chemotherapy and Immunotherapy. Int. J. Mol. Sci..

[38] Garcia J., Hurwitz H.I., Sandler A.B., Miles D., Coleman R.L., Deurloo R., Chinot O.L. (2020). Bevacizumab (Avastin(R)) in cancer treatment: A review of 15 years of clinical experience and future outlook. Cancer Treat. Rev..

[39] Zelnak A.B., O’Regan R.M. (2007). Targeting angiogenesis in advanced breast cancer. BioDrugs.

[40] Lammers P.E., Horn L. (2013). Targeting angiogenesis in advanced non-small cell lung cancer. J. Natl. Compr. Cancer Netw..

[41] De Paulis A., Prevete N., Rossi F.W., Rivellese F., Salerno F., Delfino G., Liccardo B., Avilla E., Montuori N., Mascolo M. (2009). Helicobacter pylori Hp(2–20) promotes migration and proliferation of gastric epithelial cells by interacting with formyl peptide receptors in vitro and accelerates gastric mucosal healing in vivo. J. Immunol..

[42] Honey K. (2004). TLR ligands from the natural world. Nat. Rev. Immunol..

[43] Lee H.J., Park M.K., Lee E.J., Lee C.H. (2013). Resolvin D1 inhibits TGF-beta1-induced epithelial mesenchymal transition of A549 lung cancer cells via lipoxin A4 receptor/formyl peptide receptor 2 and GPR32. Int. J. Biochem. Cell Biol..

[44] Sulciner M.L., Serhan C.N., Gilligan M.M., Mudge D.K., Chang J., Gartung A., Lehner K.A., Bielenberg D.R., Schmidt B., Dalli J. (2018). Resolvins suppress tumor growth and enhance cancer therapy. J. Exp. Med..

[45] Trombetta A., Maggiora M., Martinasso G., Cotogni P., Canuto R.A., Muzio G. (2007). Arachidonic and docosahexaenoic acids reduce the growth of A549 human lung-tumor cells increasing lipid peroxidation and PPARs. Chem. Biol. Interact..

[46] Guo Y., Nie D. (2014). Tumor-suppressing 15-lipoxygenase-2: Time for prime time?. Cell Cycle.

[47] Moussalli M.J., Wu Y., Zuo X., Yang X.L., Wistuba I.I., Raso M.G., Morris J.S., Bowser J.L., Minna J.D., Lotan R. (2011). Mechanistic contribution of ubiquitous 15-lipoxygenase-1 expression loss in cancer cells to terminal cell differentiation evasion. Cancer Prev. Res..

[48] Cherfils-Vicini J., Platonova S., Gillard M., Laurans L., Validire P., Caliandro R., Magdeleinat P., Mami-Chouaib F., Dieu-Nosjean M.C., Fridman W.H. (2010). Triggering of TLR7 and TLR8 expressed by human lung cancer cells induces cell survival and chemoresistance. J. Clin. Investig..

[49] Chatterjee S., Crozet L., Damotte D., Iribarren K., Schramm C., Alifano M., Lupo A., Cherfils-Vicini J., Goc J., Katsahian S. (2014). TLR7 promotes tumor progression, chemotherapy resistance, and poor clinical outcomes in non-small cell lung cancer. Cancer Res..

[50] Dajon M., Iribarren K., Petitprez F., Marmier S., Lupo A., Gillard M., Ouakrim H., Victor N., Vincenzo D.B., Joubert P.E. (2019). Toll like receptor 7 expressed by malignant cells promotes tumor progression and metastasis through the recruitment of myeloid derived suppressor cells. Oncoimmunology.

[51] Bauer A.K., Upham B.L., Rondini E.A., Tennis M.A., Velmuragan K., Wiese D. (2017). Toll-like receptor expression in human non-small cell lung carcinoma: Potential prognostic indicators of disease. Oncotarget.

[52] Bhagwani A., Thompson A.A.R., Farkas L. (2020). When Innate Immunity Meets Angiogenesis-The Role of Toll-Like Receptors in Endothelial Cells and Pulmonary Hypertension. Front. Med..

[53] Clark A.R., Dean J.L., Saklatvala J. (2009). The p38 MAPK pathway mediates both antiinflammatory and proinflammatory processes: Comment on the article by Damjanov and the editorial by Genovese. Arthritis Rheum..

[54] Gao P., Niu N., Wei T., Tozawa H., Chen X., Zhang C., Zhang J., Wada Y., Kapron C.M., Liu J. (2017). The roles of signal transducer and activator of transcription factor 3 in tumor angiogenesis. Oncotarget.

[55] Valle-Mendiola A., Soto-Cruz I. (2020). Energy Metabolism in Cancer: The Roles of STAT3 and STAT5 in the Regulation of Metabolism-Related Genes. Cancers.

[56] Zhang H.F., Lai R. (2014). STAT3 in Cancer-Friend or Foe?. Cancers.

[57] Larange A., Antonios D., Pallardy M., Kerdine-Romer S. (2009). TLR7 and TLR8 agonists trigger different signaling pathways for human dendritic cell maturation. J. Leukoc. Biol..

[58] Frega G., Wu Q., Le Naour J., Vacchelli E., Galluzzi L., Kroemer G., Kepp O. (2020). Trial Watch: Experimental TLR7/TLR8 agonists for oncological indications. Oncoimmunology.

[59] Vinod N., Hwang D., Azam S.H., Van Swearingen A.E.D., Wayne E., Fussell S.C., Sokolsky-Papkov M., Pecot C.V., Kabanov A.V. (2020). High-capacity poly(2-oxazoline) formulation of TLR 7/8 agonist extends survival in a chemo-insensitive, metastatic model of lung adenocarcinoma. Sci. Adv..

[60] Morra F., Luise C., Visconti R., Staibano S., Merolla F., Ilardi G., Guggino G., Paladino S., Sarnataro D., Franco R. (2015). New therapeutic perspectives in CCDC6 deficient lung cancer cells. Int. J. Cancer.

[61] Cerrato A., Morra F., Di Domenico I., Celetti A. (2019). NSCLC Mutated Isoforms of CCDC6 Affect the Intracellular Distribution of the Wild Type Protein Promoting Cisplatinum Resistance and PARP Inhibitors Sensitivity in Lung Cancer Cells. Cancers.

[62] Hung P.F., Hong T.M., Chang C.C., Hung C.L., Hsu Y.L., Chang Y.L., Wu C.T., Chang G.C., Chan N.L., Yu S.L. (2019). Hypoxia-induced Slug SUMOylation enhances lung cancer metastasis. J. Exp. Clin. Cancer Res..

[63] Gambardella J., De Rosa M., Sorriento D., Prevete N., Fiordelisi A., Ciccarelli M., Trimarco B., De Luca N., Iaccarino G. (2018). Parathyroid Hormone Causes Endothelial Dysfunction by Inducing Mitochondrial ROS and Specific Oxidative Signal Transduction Modifications. Oxid. Med. Cell. Longev..

[64] Liotti F., De Pizzol M., Allegretti M., Prevete N., Melillo R.M. (2017). Multiple anti-tumor effects of Reparixin on thyroid cancer. Oncotarget.

[65] Pellet-Many C. (2015). Chemotactic Migration of Endothelial Cells Towards VEGF-A(1)(6)(5). Methods Mol. Biol..

[66] Liotti F., Collina F., Pone E., La Sala L., Franco R., Prevete N., Melillo R.M. (2017). Interleukin-8, but not the Related Chemokine CXCL1, Sustains an Autocrine Circuit Necessary for the Properties and Functions of Thyroid Cancer Stem Cells. Stem Cells.

[67] Collina F., La Sala L., Liotti F., Prevete N., La Mantia E., Chiofalo M.G., Aquino G., Arenare L., Cantile M., Liguori G. (2019). Is a Novel Predictive Factor and Therapeutic Target for Radioactive Iodine Refractory Thyroid Cancer. Cancers.

[68] Morra F., Merolla F., Criscuolo D., Insabato L., Giannella R., Ilardi G., Cerrato A., Visconti R., Staibano S., Celetti A. (2019). CCDC6 and USP7 expression levels suggest novel treatment options in high-grade urothelial bladder cancer. J. Exp. Clin. Cancer Res..

