# Supplementary material for: Toll-Like Receptor 7 Mediates Inflammation Resolution and Inhibition of Angiogenesis in Non-Small Cell Lung Cancer
Source: Cancers (Basel). 2021 Feb 10;13(4):740. doi: 10.3390/cancers13040740 (PMC7916730; doi:10.3390/cancers13040740)
Supplement: Supplementary file 1 [file cancers-13-00740-s001.pdf]

cancers

MDPI

Supplementary Materials: Toll-Like Receptor 7 Mediates Inflammation Resolution and Inhibition of Angiogenesis in Non-Small Cell Lung Cancer

Federica Liotti, Maria Marotta, Daniela Sorriento, Emanuela Pone, Francesco Morra, Rosa Marina Melillo and Nella Prevete

Supplementary Methods

Tumorigenicity in Immunocompromised Mice

Each group of 8 mice (4-week-old CD1 nu/nu females) was inoculated subcutaneously with A549 transfected with control shRNAs (shCTR) or shTLR7 (5×10^6 cells/mouse) for in vivo studies. These experiments were conducted according to Italian regulations for experimentation on animals.

A

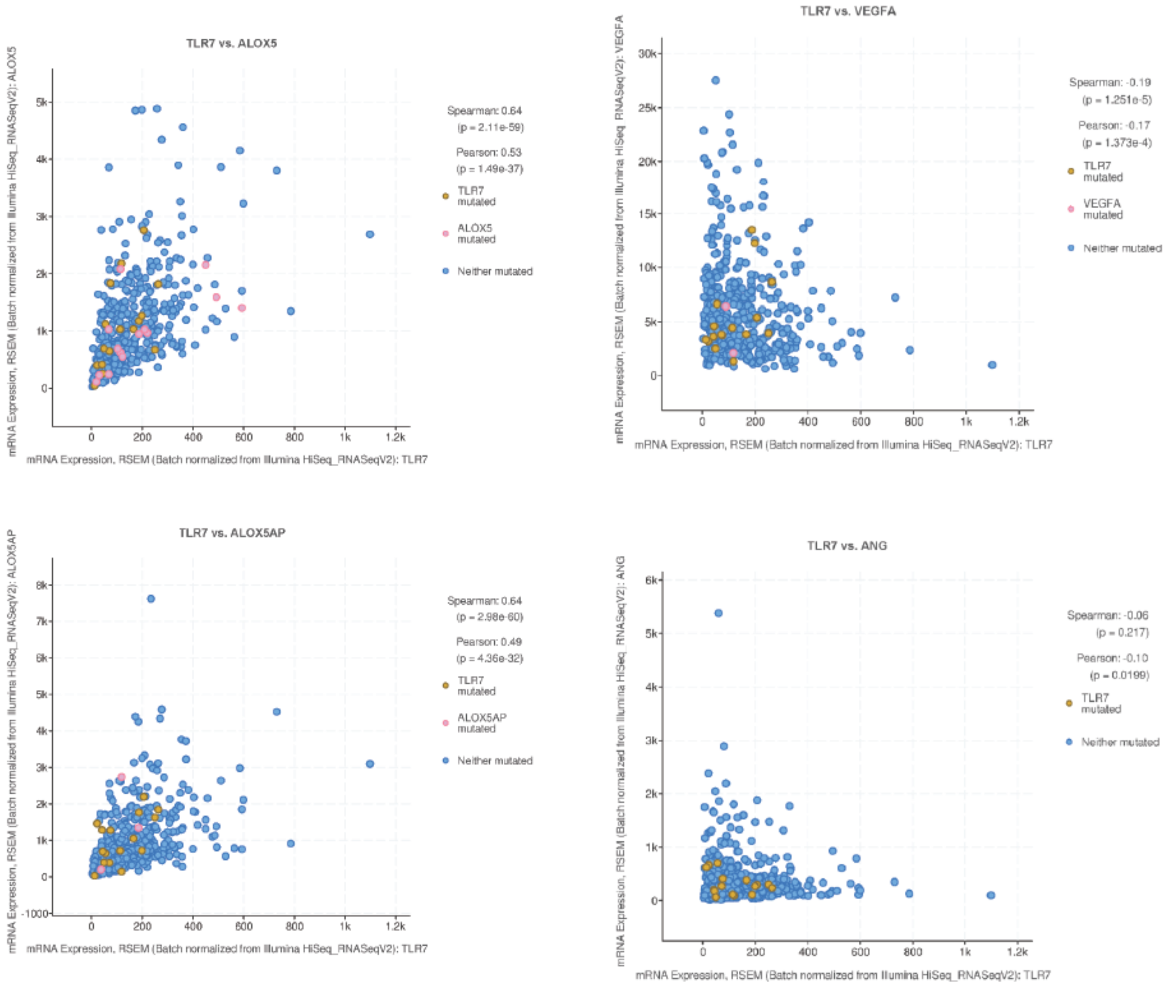

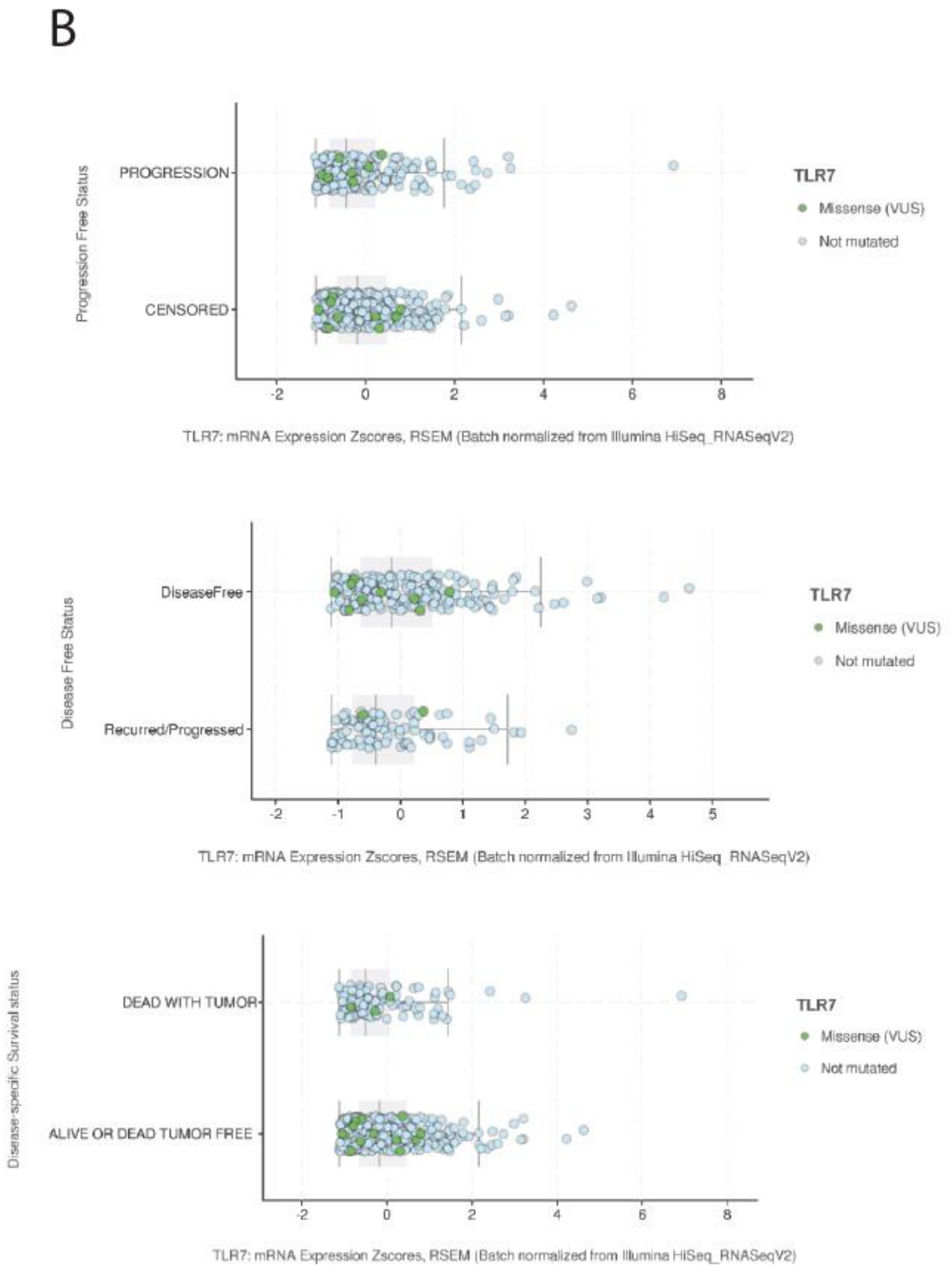

Figure S1. TLR7 correlation to NSCLC patients' characteristics. (**A**) Correlation between the mRNA expression levels of the indicated markers in 503 patients affected by NSCLC (adenocarcinoma). Spearman factor, Pearson factor and the relative p are indicated. (**B**) TLR7 mRNA expression levels of 503 patients affected by NSCLC (adenocarcinoma) stratified for progression free, disease-free and disease-specific survival status.

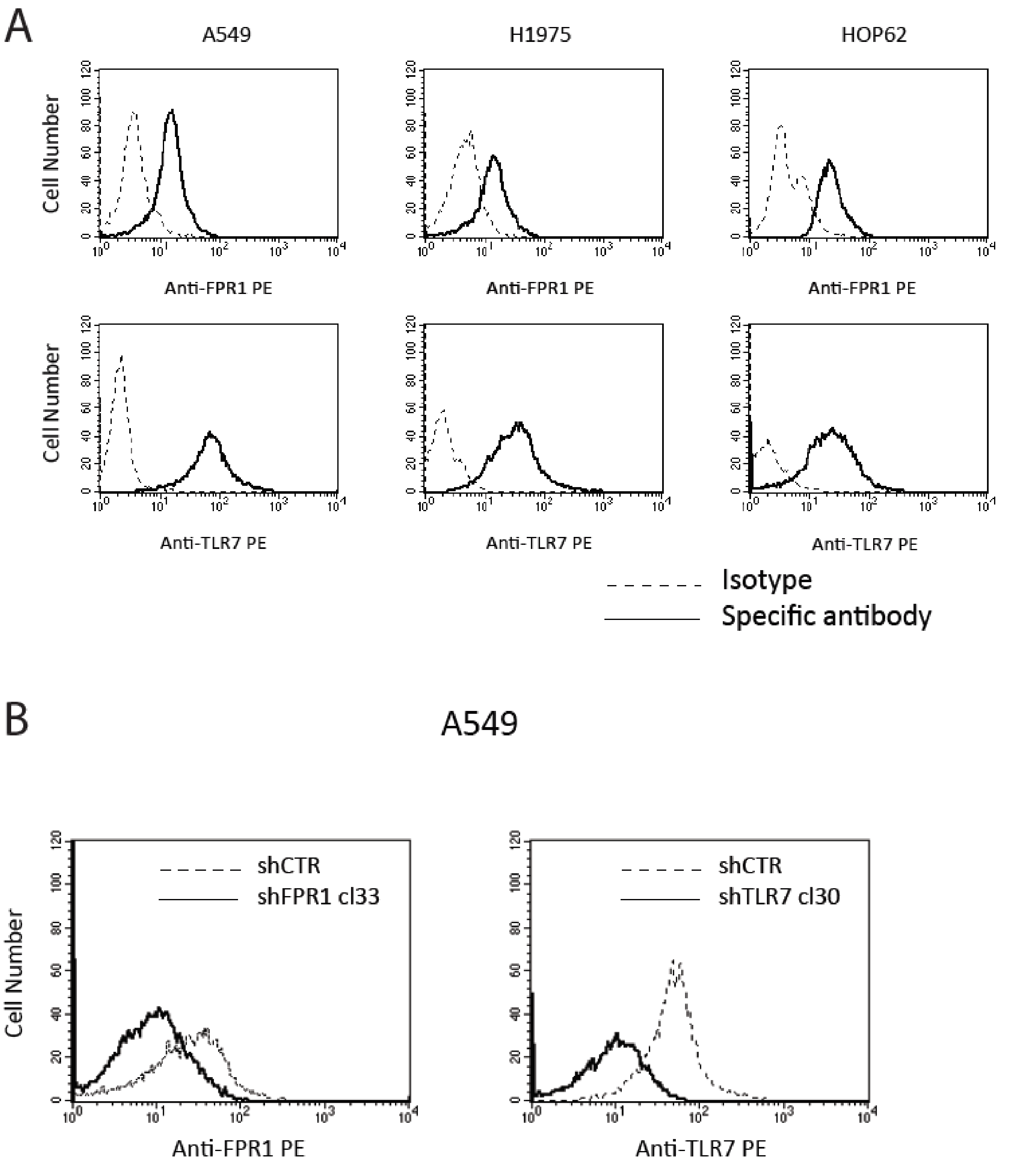

Figure S2. FPR1 and TLR7 expression in NSCLC cells. (**A**) FPR1 and TLR7 protein expression levels (mean fluorescence intensity), assessed by cytofluorimetric analysis, in A549, H1975 and HOP62 cells. A representative experiment is shown. (**B**) FPR1 and TLR7 protein expression levels (mean fluorescence intensity), assessed by cytofluorimetric analysis, in A549 shCTR, shFPR1, and shTLR7. A representative clone is shown.

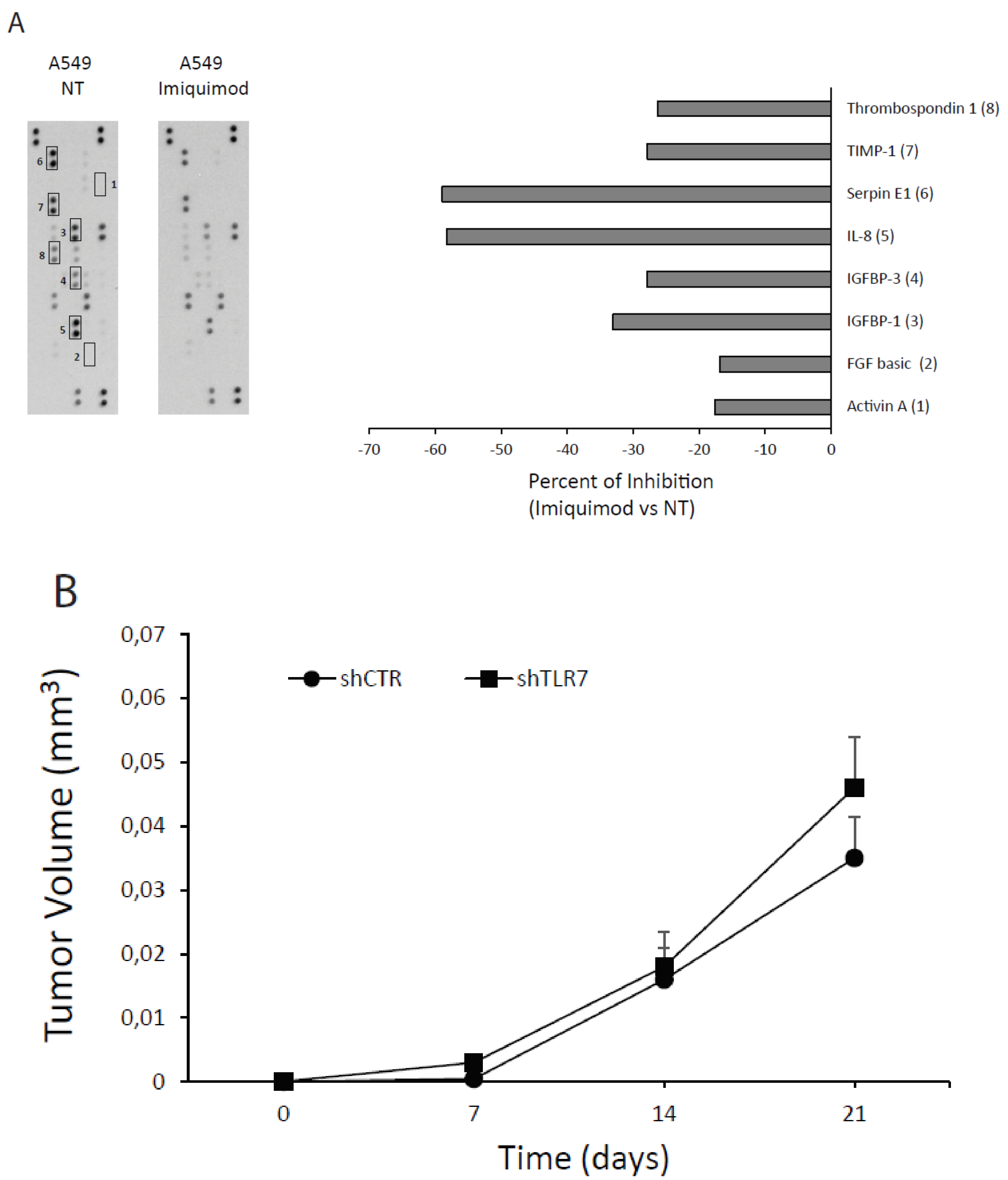

Figure S3. Effects of TLR7 modulation on NSCLC angiogenic and tumorigenic potential. (**A**) Analysis of proteins in CM from A549 treated or not with Imiquimod (1 µg/mL) using angiogenesis associated protein antibody arrays. The array images and the relative quantitative profiles of protein levels are shown. (**B**) Xenograft growth curves of A549 cells stably silenced for TLR7 (shTLR7-2 clones) or control cells (shCTR-a mass population) in immunocompromised mice.

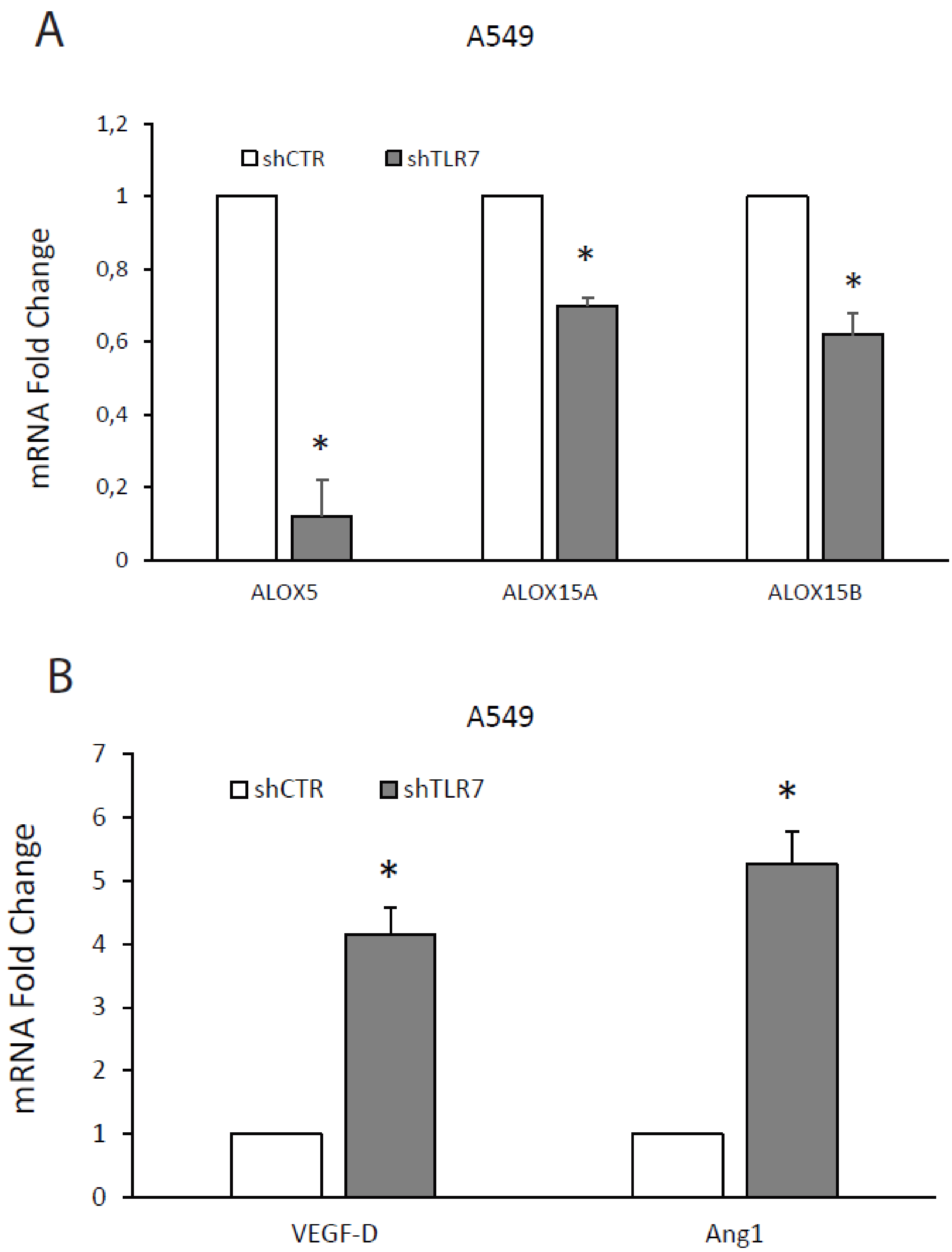

Figure S4. Effects of TLR7 silencing on SPM biosynthesis machinery and angiogenic potential of NSCLC cells. (**A**) ALOX5, ALOX15A, ALOX15B mRNA expression in A549 cells stably silenced for TLR7. (shTLR7, 2 clones) and the relative control (shCTR, a mass population). Data are represented as mean ± SD of 5 independent experiments. * $P < 0.05$ compared to the control. (**B**) VEGF-D and Ang1 mRNA expression in A549 cells stably silenced for TLR7 (shTLR7, 2 clones) and the relative control (shCTR, a mass population). Data are represented as mean ± SD of 5 independent experiments. * $P < 0.05$ compared to the control.

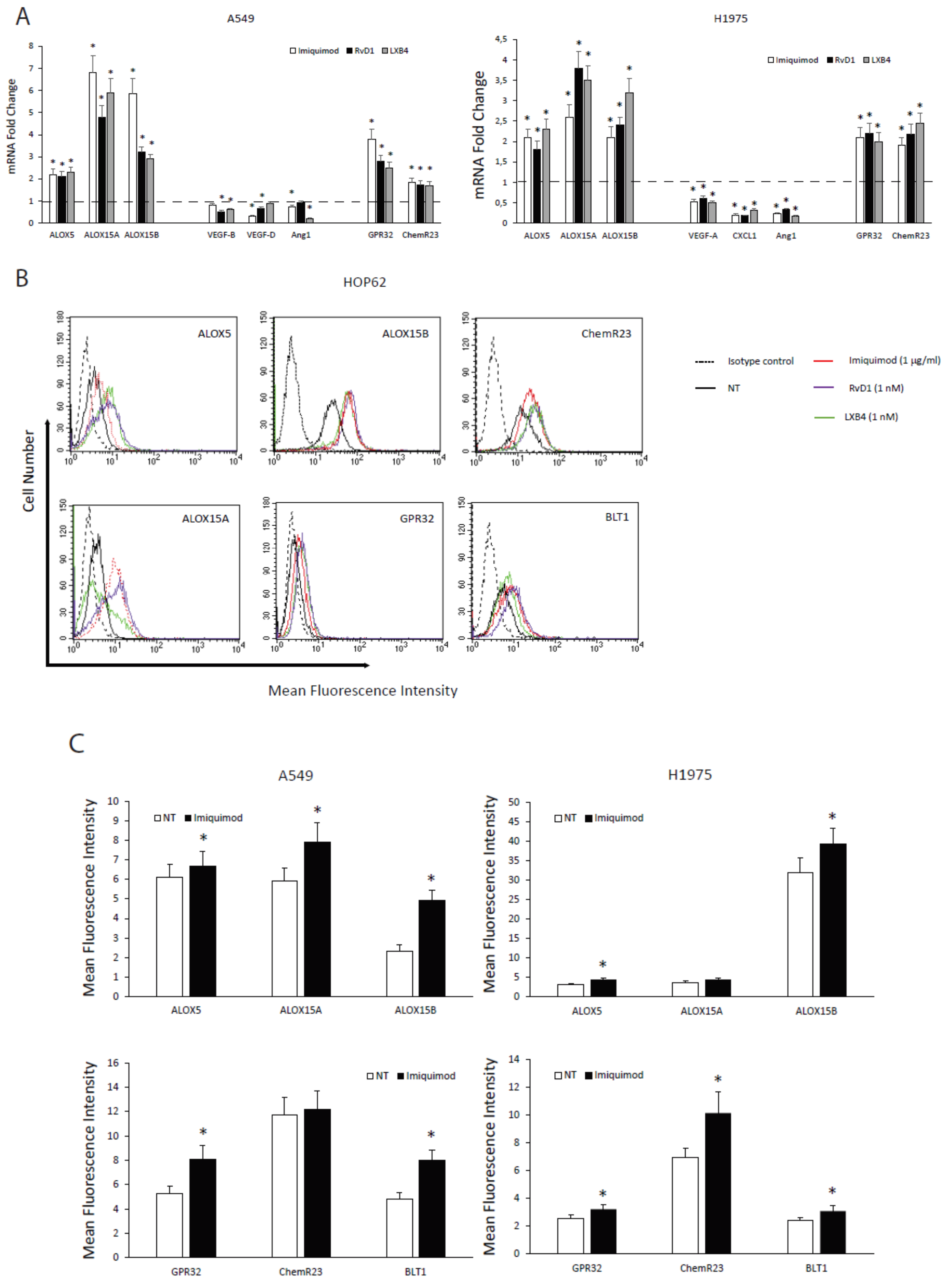

Figure S5. Effects of TLR7 activation on SPM biosynthetic machinery and angiogenic potential of NSCLC cells. (**A**) ALOX5, ALOX15A, ALOX15B, VEGF-A, VEGF-B, VEGF-D, CXCL1, Ang1, GPR32 and ChemR23 mRNA fold change in A549 or H1975 cells treated with Imiquimod (1 μg/mL), RvD1 (1 nM), or LXB4 (1 nM) for 3 h. Data are represented as mean ± SD of 5 independent experiments. * $P < 0.05$ compared to the control (dotted line). (**B**) ALOX5, ALOX15A, ALOX15B, GPR32, ChemR23 and BLT1 protein expression levels (mean fluorescence intensity), assessed by cytofluorimetric analysis, in HOP62 cells treated with Imiquimod (1 μg/mL), RvD1 (1 nM), or LXB4 (1 nM) for 6 h. A representative experiment is

shown. (**C**) ALOX5, ALOX15A, ALOX15B, GPR32, ChemR23 and BLT1 protein expression levels (mean fluorescence intensity), assessed by cytofluorimetric analysis, in A549 and H1975 cells treated with Imiquimod (1 µg/mL) for 6 h. Data are represented as mean ± SD of 3 independent experiments. * *P* < 0.05 compared to the control (NT).

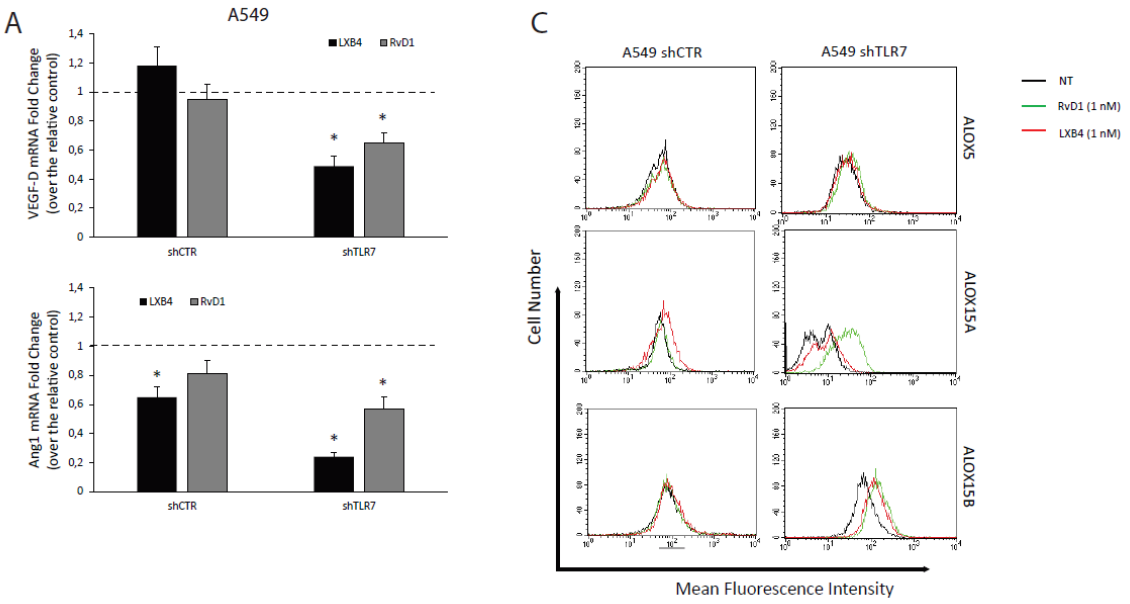

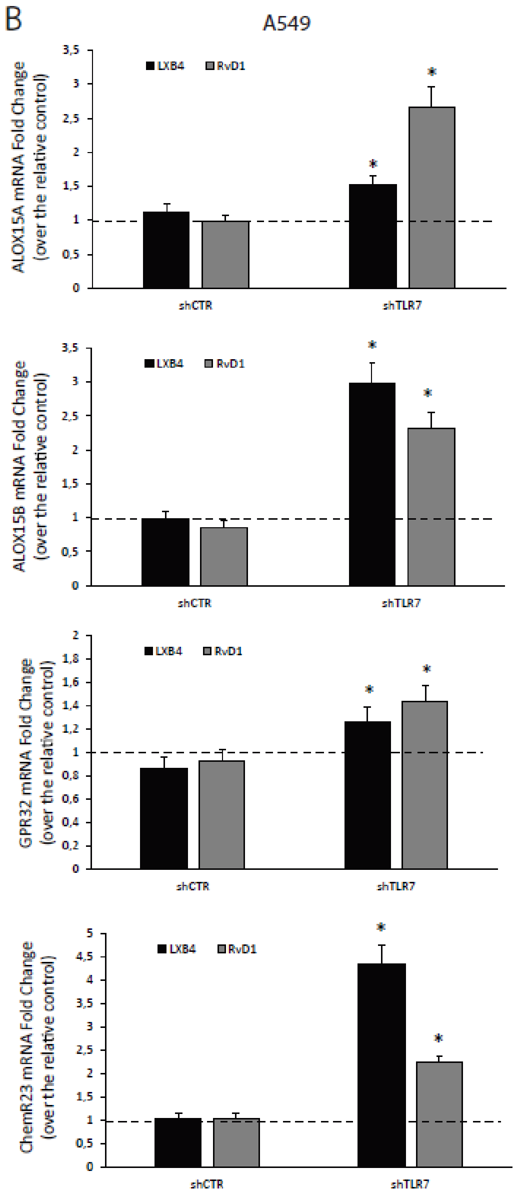

Figure S6. Anti-angiogenic and pro-resolving effects of SPMs in NSCLC cells. (A) mRNA fold change for VEGF-D and Ang1 over control induced by LXB4 (1 nM) or RvD1 (1 nM) in A549 shCTR (a mass population) and shTLR7 (2 clones) (3 h treatment). Data are represented as mean ± SD of 5 independent experiments. * $P < 0.05$ compared to the control (dotted line). (**B**) mRNA fold change for ALOX15A, ALOX15B, GPR32, and ChemR23 over control induced by LXB4 (1 nM) or RvD1 (1 nM) in A549 shCTR (a mass population) and shTLR7 (2 clones) (3 h treatment). Data are represented as mean ± SD of 5 independent experiments. * $P < 0.05$ compared to the control (dotted line). (**C**) ALOX5, ALOX15A, and ALOX15B protein expression levels (mean fluorescence intensity), assessed by cytofluorimetric analysis, in A549 shCTR and shTLR7 cells treated with RvD1 (1 nM) or LXB4 (1 nM) for 6 h. A representative experiment is shown.

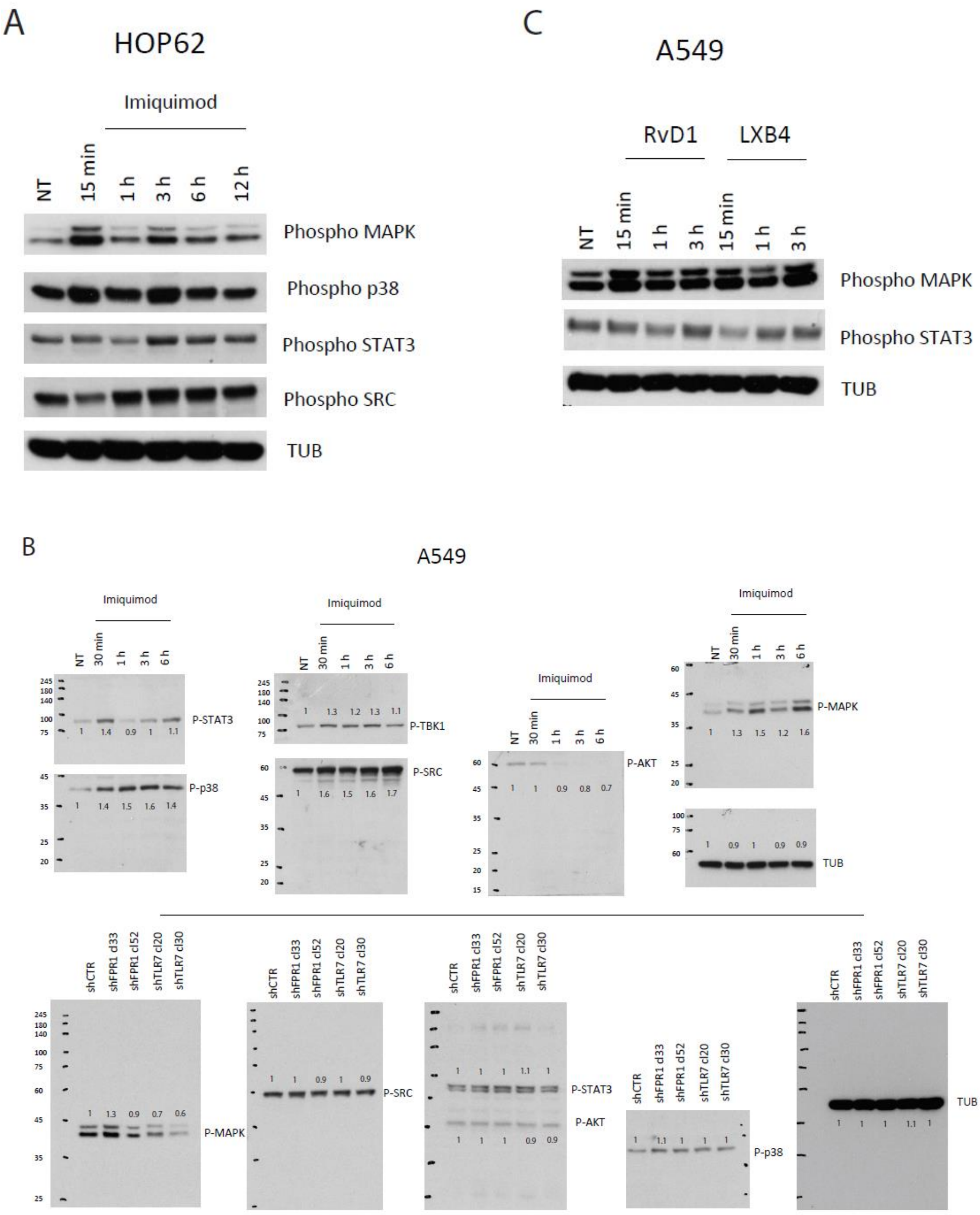
A
HOP62
Imiquimod
NT
15 min
1 h
3 h
6 h
12 h
Phospho MAPK
Phospho p38
Phospho STAT3
Phospho SRC
TUB
C
A549
RvD1
LXB4
NT
15 min
1 h
3 h
15 min
1 h
3 h
Phospho MAPK
Phospho STAT3
TUB
B
A549
Imiquimod
NT
30 min
1 h
3 h
6 h
P-STAT3
P-p38
P-TBK1
P-SRC
P-AKT
P-MAPK
TUB
shCTR
shFPR1 cl33
shFPR1 cl52
shTLR7 cl20
shTLR7 cl30
P-MAPK
P-SRC
P-STAT3
P-AKT
P-p38
TUB

	pMAPK	pAKT	pSTAT3	pSRC	p-p38
NT	1,0	1,0	1,0	1,0	1,0
Imiquimod 30 min	1,6	1,1	1,5	1,5	1,1
Imiquimod 1 h	1,6	1,0	1,0	1,6	1,3
Imiquimod 3 h	1,4	0,8	1,3	1,4	1,6
Imiquimod 6 h	1,0	0,6	1,1	1,7	0,8

	pMAPK	pSTAT3	p-p38	pSRC
shCTR	1,0	1,0	1,0	1,0
shFPR1 cl 33	1,5	1,1	1,3	1,0
shFPR1 cl 52	1,0	1,0	1,3	1,0
shTLR7 cl 20	0,1	1,0	1,1	1,1
shTLR7 cl 30	0,2	1,1	1,3	1,2

	pMAPK	pAKT	pSTAT3	pSRC	p-p38
NT	1,0	1,0	1,0	1,0	1,0
Imiquimod 30 min	1,5	0,9	1,3	1,2	1,4
Imiquimod 1 h	1,6	0,9	1,1	1,1	1,1
Imiquimod 3 h	1,4	0,8	1,3	1,0	1,4
Imiquimod 6 h	0,9	1,1	1,6	1,4	1,1

	pMAPK	pSTAT3	p-p38	pSRC
shCTR	1,0	1,0	1,0	1,0
shFPR1 cl 33	1,1	1,0	1,2	1,2
shFPR1 cl 52	0,9	1,0	1,2	1,1
shTLR7 cl 20	0,6	1,1	1,0	1,1
shTLR7 cl 30	0,5	1,1	1,1	1,2

Figure S7. Signaling pathways involved in anti-angiogenic and pro-resolving functions of TLR7. (**A**) Activation kinetics of MAPK, p38, STAT3 and SRC in HOP62 cells treated or not for the indicated time points with Imiquimod (1 μg/mL), assessed by western blot for their phosphorylated forms. α-tubulin (TUB) was used as normalization marker. A representative experiment is shown. (**B**) Activation kinetics of MAPK, AKT, STAT3, SRC, p38 and TKB1 in A549 cells treated or not for the indicated time points with Imiquimod (1 μg/mL), assessed by western blot for their phosphorylated forms. α-tubulin (TUB) was used as normalization marker. Intensity ratio of three independent experiments is shown. Constitutive MAPK, AKT, STAT3, SRC, and p38 activation levels in A549 shCTR (a mass population), shFPR1 (clones 33 and 52), and shTLR7 (clones 20 and 30) assessed by western blot for their phosphorylated forms. α-tubulin (TUB) was used as normalization marker. The intensity ratio of three independent experiments is shown. (**C**) Activation kinetics of MAPK and STAT3 in A549 cells treated or not for the indicated time points with RvD1 (1 nM) or LXB4 (1 nM), assessed by western blot for their phosphorylated forms. α-tubulin (TUB) was used as normalization marker. A representative experiment is shown.

Table S1. List of primers; F- Forward, R-Reverse in 5′-3′ direction.

GPR32 F	ACTATATTGTCTCCAGGCAGTG
GPR32 R	ACAGTGCGGTGGTTCAGG
ChemR23 F	CTGTCCACACCTGGGTCTTC
ChemR23 R	CCCCACAGGGTCCATTTGG
BLT-1 F	GCCCTGGAAAACGAACATGA
BLT-1 R	TTAGATGGAAGGCCCGGTG
VEGF-A F	GTGAATGCAGACCAAAGAAAG
VEGF-A R	AAACCCTGAGGGAGGCTC
VEGF-B F	TGTCCCTGGAAGAACACAGCC
VEGF-B R	GCCATGTGTCACCTTCGCA
VEGF-C F	ATGTTTTCCTCGGATGCTGGA
VEGF-C R	CATTGGCTGGGGAAGAGTTT
VEGF-D F	GTATGGACTCTCGCTCAGCAT
VEGF-D R	AGGCTCTCTTCATTGCAACAG
ANG1 F	CCTCGCTGCCATTCTGACTC
ANG1 R	ACTCTCACGACAGTTGCCATC
CXCL1 F	CACCATGGCCCGCGCTGCTCTC
CXCL1 R	GTTGGATTTGTCACTGTT
ALOX15A F	GACTTTGAGGTTTCGCTGGC
ALOX15A R	GACCACACCAGAAAATCCGG
ALOX15B F	GAGGGTACAGCCAAGGTGTC
ALOX15B R	AAAGCAGAGCCAGCCTGTAG
ALOX5 F	AGTCCTCAGGCTTCCCCAAGT
ALOX5R	CATGCCCAGGAACAGCTCGTT
β-ACT F	TGCGTGACATTAAGGAGAAG
β-ACT R	GCTCGTAGCTCTTCTCCA
